# Quantum thermalization must occur in translation-invariant systems at high temperature

**DOI:** 10.1038/s41467-025-66777-7

**Published:** 2025-12-16

**Authors:** Saúl Pilatowsky-Cameo, Soonwon Choi

**Affiliations:** Center for Theoretical Physics, Massachusetts Institute of Technology, Cambridge, MA USA

**Keywords:** Quantum mechanics, Statistical physics, Quantum information

## Abstract

Quantum thermalization describes how closed quantum systems can effectively reach thermal equilibrium, resolving the apparent incongruity between the reversibility of Schrödinger’s equation and the second law of thermodynamics. Despite its ubiquity and conceptual significance, the precise conditions that give rise to quantum thermalization are still not well understood. After nearly a century of efforts, we have yet to find a complete mathematical proof that an effective statistical description naturally emerges the underlying quantum dynamics in generic settings. Here, we prove that quantum thermalization must occur in any qubit system with local interactions under three conditions: (i) high effective temperature, (ii) translation invariance, and (iii) no perfect resonances in the energy spectrum. Specifically, we show that a typical, low-complexity pure state drawn from any ensemble with large entropy and well-defined effective temperature becomes locally indistinguishable from a Gibbs state upon unitary evolution. In this setting, our rigorous results prove the widely anticipated notion that statistical physics should be understood as an emergent phenomenon, explicitly derived from the first principles of quantum mechanics.

## Abstract (web summary)

Explaining how reversible quantum dynamics give rise to thermal behavior is a century-old problem in statistical physics. This work provides a mathematical proof that quantum thermalization must occur in generic, translation-invariant systems at high temperature.

## Introduction

Establishing a rigorous mathematical foundation for quantum thermalization^1–5^ is arguably the most important challenge in quantum statistical physics. Such a feat would position the foundational principle of statistical mechanics—that a system evolves into a state with maximum entropy—as a derived, provable statement. The significance extends beyond this fundamental theory, as quantum thermalization underpins key questions across many other branches of physics, including the equilibration of heavy-ion experiments described by quantum chromodynamics^6^ and the formation of black holes in certain quantum-gravity theories^7^. It also relates to practical applications, such as the development of passive quantum memories for quantum computation^8^.

The emergence of statistical mechanics in quantum systems is a century-old problem^9^ which has been attacked by a multitude of complementary approaches, including devising conjectures such as the eigenstate thermalization hypothesis (ETH)^10–12^ and performing numerical or quantum simulations^13–19^. While these approaches provide useful insights and relevant conceptual understanding, they do not constitute the rigorous approach desired to tackle foundational questions. Recent developments in quantum information theory present us with a new avenue. By leveraging symmetries and information theory, a number of rigorous and precise statements about quantum dynamics have been formulated^20–33^, bringing us a step closer to a complete proof of quantum thermalization.

Here, we adopt such a rigorous, information-theoretic approach to identify a broad set of physical conditions under which quantum thermalization must provably occur, without appealing to any unproven hypothesis. We prove that quantum thermalization must occur in any qubit system with local interactions under three conditions: high effective temperature, translation invariance, and no perfect resonances in the energy spectrum. Unlike previous results, we prove this statement without breaking the locality of the Hamiltonian^33^, imposing any assumptions on the energy distribution of the initial state^27,30^, or relying on empirical postulates such as the ETH or the emergence of typicality in dynamics^22^.

## Results

### Quantum thermalization

**Fig. 1:**
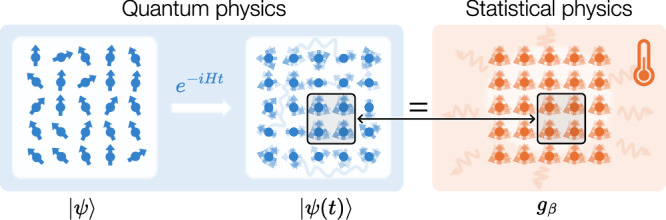
Main idea of quantum thermalization. The local indistinguishability between time-evolved quantum many-body states (left) and the thermal Gibbs states (right) establishes the bridge between quantum and statistical physics.

We begin by formulating the precise statement of quantum thermalization. In this work, we consider a large but limited class of quantum systems: *N* qubits arranged on a periodic lattice in arbitrary spatial dimension *D*, with dynamics described by a local, translation-invariant time-independent Hamiltonian *H*. Once the system is initialized in a pure state $\left\vert \psi \right\rangle$ that is far from equilibrium, quantum thermalization dictates that upon unitary time evolution, the state

1 $$\left\vert \psi (t)\right\rangle={e}^{-iHt/\hslash }\left\vert \psi \right\rangle$$

becomes locally indistinguishable from a Gibbs state

2 $${g}_{\beta }=\frac{{e}^{-\beta H}}{{{{\rm{tr}}}}({e}^{-\beta H})},$$

at a corresponding inverse temperature *β* = 1/(*k*_*B*_*T*) determined by the conservation of global energy $\langle \psi| H|\psi \rangle={{{\rm{tr}}}}({g}_{\beta }H)$ (in the presence of further conservation laws, *g*_*β*_ may be replaced by a generalized Gibbs state). This process is depicted in Fig. 1. Hereafter, we work with units where the Boltzmann constant *k*_*B*_ and the Planck constant *ℏ* equal 1.

Local indistinguishability means equality of local measurement outcomes. Specifically, for any geometrically local and finite region *A* and for any typical late time *t*, we demand that the evolved state reproduces the thermal expectation values of any observable *O*_*A*_ supported in *A*,

3 $$\langle \psi (t)| {O}_{A}| \psi (t)\rangle={{{\rm{tr}}}}({g}_{\beta }{O}_{A})\pm \varepsilon (N),$$

where *ε*(*N*) accounts for finite-size corrections^34,35^, which must vanish in the thermodynamic limit *N* → *∞*. Equivalently, we write

4 $${\parallel {g}_{\beta }-\psi(t)\parallel}_{A} \le \varepsilon (N),$$

where we introduced the *local trace norm* ∥ ⋅ ∥_*A*_, which measures the distinguishability between *g*_*β*_ and $\psi(t)=\vert \psi (t)\rangle \langle \psi (t)\vert$ to an optimal observable in region *A*, i.e., ${\parallel {g}_{\beta }-\psi (t){\parallel }}_{A}={\max }_{{O}_{A}}\frac{1}{\parallel {O}_{A}{\parallel }_{{{{\rm{op}}}}}}| {{{\rm{tr}}}}(({g}_{\beta }-\psi (t)){O}_{A})|$. We say the state *ψ* thermalizes under *H* if Eq. (4) holds.

To establish a rigorous and universal statement about quantum thermalization, one needs to take into account two important considerations. First, thermalization does not occur in all systems; many examples of non-thermalizing dynamics have been recognized over the past decades^36–40^, and the precise conditions that ensure thermalization or the lack thereof have not been completely identified. It turns out, as we show here, that only one extra condition on top of translation invariance suffices to guarantee thermalization of typical states: the energy spectrum {*E*_1_, *E*_2_, *E*_3_, …} has nondegenerate gaps, meaning that *E*_*i*_ + *E*_*j*_ = *E*_*k*_ + *E*_*l*_ is exactly satisfied only if (*i*, *j*) = (*k*, *l*) or (*i*, *j*) = (*l*, *k*). This condition is conjectured to hold for all but a fine-tuned (measure-zero) subset of Hamiltonians, as we further discuss later. Violations of this condition imply that one can harvest (potentially hidden) pairs of noninteracting qubit degrees of freedom in the system (see Supplementary Note), which is not expected for ergodic systems. To the best of our knowledge, variants of the nondegenerate gap condition have been relied on by all prior works that rigorously prove quantum equilibration^23,24,27,31^.

The second consideration is that it is provably impossible to devise a procedure (either formulated as a general theorem or computer algorithm) that determines whether a specific state thermalizes under a given translation-invariant Hamiltonian—such a task has been shown to be computationally undecidable^41^. Therefore, we focus on establishing a probabilistic statement. We say an ensemble ${{{\mathcal{E}}}}$ consisting of pure states thermalizes if

5 $${\mathbb{E}}_{\psi} \,{\mathbb{E}}_{t}\left[{\parallel {g}_{\beta }-\psi (t){\parallel }}_{A}\right]\le \varepsilon (N),$$

where the average ${\mathbb{E}}_{\psi}$ is taken over initial states *ψ* sampled from the ensemble ${{{\mathcal{E}}}}$ and ${{\mathbb{E}}_t}[\,\cdot \,]={\lim }_{T\to \infty }\frac{1}{T}\int_{0}^{T}{{{\rm{d}}}}t[\,\cdot \,]$ denotes averaging over evolution times *t* ≥ 0. The local trace norm being averaged is nonnegative, and therefore Eq. (5) implies quantum thermalization of all typical initial states $\psi \in {{{\mathcal{E}}}}$ and evolution times *t*.

An important question is to identify for which classes of initial-state ensembles one can prove a meaningful statement about thermalization. Generally, we wish to choose the ensemble as large as possible for a broad applicability, yet we note that for certain ensembles, the statement of thermalization is less meaningful. For example, if ${{{\mathcal{E}}}}$ contains all states in the Hilbert space uniformly distributed, then thermalization follows trivially, simply because most states in the Hilbert space are already thermal^22^. Moreover, such random pure states are not physically realizable either in the laboratory or nature due to their exponentially large complexity^42^. Instead, the interesting scenario is to consider out-of-equilibrium states that can be easily prepared. In this direction, a recent work has shown the thermalization of the ensemble of random product states in 1D^31^. While an important milestone, this result only concerns the infinite effective temperature regime *β* = 0, wherein the “thermal” state is independent of the choice of Hamiltonian and energy conservation does not play an important role.

In order to draw a physically meaningful statement about quantum thermalization at finite temperature, we introduce a class of ensembles ${{{{\mathcal{E}}}}}_{\beta,{{{\mathcal{C}}}}}$ with two parameters *β* and ${{{\mathcal{C}}}}$ satisfying the following three properties:

- (P1) *Shallow complexity *${{{\mathcal{C}}}}$: any state in ${{{{\mathcal{E}}}}}_{\beta,{{{\mathcal{C}}}}}$ can be prepared from a product state by a quantum circuit of depth at most ${{{\mathcal{C}}}}$, where ${{{\mathcal{C}}}}(N)$ may grow at most subpolynomially in the system size *N*.
- (P2) *Inverse temperature β*: the average global energy matches the thermal value ${\mathbb{E}}_{\psi} [{{{\rm{tr}}}}(\psi H)]={{{\rm{tr}}}}({g}_{\beta }H)$.
- (P3) *Maximally entropic ensemble* (MEE): the von Neumann entropy of the average state $\rho={\mathbb{E}}_{\psi} [\psi ]$ is maximal among all other ensembles with the same complexity (P1) and temperature (P2).

Property (P1) guarantees that any state in the ensemble is far from equilibrium and physically relevant, (P2) controls the effective temperature of the ensemble, and (P3) implies that the ensemble is large, in a precise sense. Our main result establishes the quantum thermalization of any ensemble satisfying these three properties at high temperature:

**Theorem 1** (Quantum thermalization). Consider any translation invariant, geometrically local Hamiltonian for *N* qubits in *D*-dimensional lattice with nondegenerate spectral gaps. Below a certain threshold inverse temperature β_*_ > 0 (independent of *N*), any ensemble satisfying properties (P1–3) thermalizes [i.e., satisfies Eq. (5)], with finite-size corrections $\varepsilon (N)\le O\left({N}^{-1/2+\delta }\right)$ for any *δ* > 0 and geometrically local region *A* of constant size.

In particular, if we consider an ensemble of trivial complexity ${{{\mathcal{C}}}}=0$, we establish a statement about thermalization of initial product states:

**Corollary 1**. Under the conditions of Theorem 1, any maximally entropic ensemble of pure product states at inverse temperature *β* ≤ β_*_ thermalizes.

**Fig. 2:**
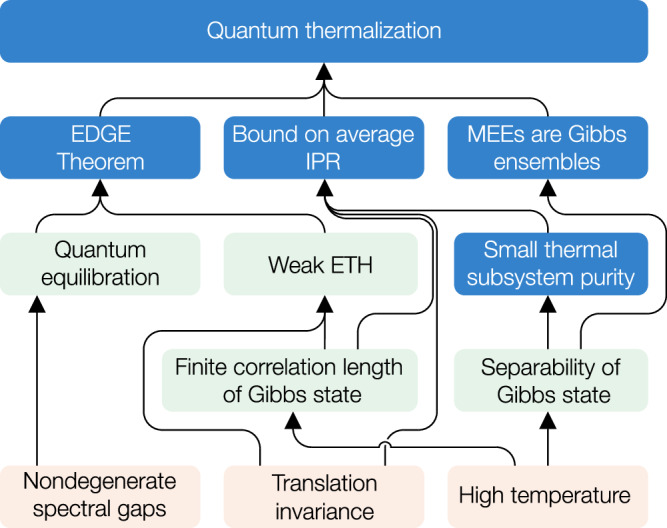
Flowchart for our proof of quantum thermalization. We summarize logical implications among our results (blue), previously known results (green), and assumptions (orange).

The proof of Theorem 1 is enabled by synthesizing a number of previously-established and newly-developed technical results, summarized by the flowchart in Fig. 2. Among them, three particular results are pivotal. First, we introduce a notion that we call *energy-dispersed Gibbs ensembles* (EDGEs)—these are ensembles of pure states, wherein individual states are typically supported in many energy eigenstates (energy dispersed), while their collection averages to the Gibbs state (Gibbs ensemble). By adapting previous results (the weak ETH^29,43–45^ and quantum equilibration theorems^24^), our EDGE Theorem shows that EDGEs thermalize under the nondegenerate spectral gaps condition, as long as the Gibbs state has a finite correlation length, which provably occurs at high enough temperature^46^$| \beta | < {\beta }_{*}^{{{{\rm{(corr)}}}}}$. Second, we note that any maximally entropic ensemble of shallow-complexity states at high temperature is a Gibbs ensemble. This follows from a recent result regarding the separability of Gibbs states^47^, which proves the existence of a Gibbs ensemble consisting of product states at sufficiently high temperature $| \beta | < {\beta }_{*}^{({{{\rm{sep}}}})}$. Finally, using the translation invariance, we show that any high-temperature Gibbs ensemble of shallow-complexity states must also be energy dispersed, and thus constitute an EDGE. We now give an overview of these results, with full technical details available in the Supplementary Note.

### EDGE theorem

We begin by formally introducing EDGEs.

**Definition 1** (Energy-dispersed Gibbs ensemble). For any *N*-qubit Hamiltonian *H* with Gibbs state *g*_*β*_, an ensemble of states ${{{{\mathcal{E}}}}}_{\beta }$ is an EDGE if it satisfies the following two conditions:

- *Gibbs ensemble*: The average of the ensemble is close to *g*_*β*_,
  6 $$\left\Vert {\mathbb{E}}_{\psi} [\psi ]-{g}_{\beta }\right\Vert \le {2}^{-N}{\varepsilon }_{{{{\rm{GE}}}}}(N),$$
  where ∥ ⋅ ∥ is the usual trace norm.
- *Energy dispersion*: The average inverse participation ratio (IPR) in the energy eigenbasis ${\{\vert {E}_{j}\rangle \}}_{j}$ is upper bounded as
  7 $${\mathbb{E}}_{\psi} \left[{\sum }_{j=1}^{{2}^{N}}{| \langle {E}_{j}| \psi \rangle | }^{4}\right]\le {\varepsilon }_{{{{\rm{ED}}}}}(N).$$

The two functions *ε*_ED_(*N*) and *ε*_GE_(*N*) should be nonnegative and vanish in the thermodynamic limit *N* → *∞*, but otherwise can be arbitrary. The dispersion in the energy eigenbasis is measured by a small IPR ∑_*j*_∣〈*E*_*j*_∣*ψ*〉∣^4^, which is the inverse of the so-called effective dimension or participation ratio, that estimates the number of eigenstates on which $\left\vert \psi \right\rangle$ is supported.

Our next result shows that typical states in an EDGE thermalize as long as the Gibbs state has a finite correlation length. A finite correlation length means that correlations between any pair of observables decay at least exponentially fast in their distance (up to a boundary-dependent factor). This condition holds below a certain constant inverse temperature ${\beta }_{*}^{{{{\rm{(corr)}}}}}$ for any local Hamiltonian in any geometry^46^ (see Supplementary Note).

**Theorem 2** (EDGE Theorem). Consider any translation-invariant, geometrically local Hamiltonian for *N* qubits in a *D*-dimensional lattice with nondegenerate spectral gaps. Let ${{{{\mathcal{E}}}}}_{\beta }$ be an EDGE and assume that the Gibbs state *g*_*β*_ has a finite correlation length. Then, for any geometrically local region *A* with a constant number of qubits *N*_*A*_,

8 $$\begin{array}{l}{\mathbb{E}}_{\psi} {\mathbb{E}}_{t}\left[{\parallel {g}_{\beta }-\psi (t){\parallel }}_{A}\right] \\ \le O({N}^{-\gamma })+2{\varepsilon }_{{{{\rm{GE}}}}}(N)+{2}^{{N}_{A}}{\varepsilon }_{{{{\rm{ED}}}}}{(N)}^{1/2},\end{array}$$

for any *γ* < 1/(*D* + 1) at any temperature and for any *γ* < 1/2 at sufficiently high temperature.

Our proof of Theorem 2 is inspired by ref. ^31^. Below, we present the key ideas, with the full technical details available in the Supplementary Note.

*Proof*. Quantum thermalization, ∥*ψ*(*t*)−*g*_*β*_∥_*A*_ → 0, can be split into two fine-grained statements. First, we show equilibration, which dictates that pure quantum states at late times become locally indistinguishable from their time-averaged mixed state ${\rho }_{\infty }={\mathbb{E}}_{\tau} [\psi (\tau )]$, i.e., ∥*ψ*(*t*)−*ρ*_*∞*_∥_*A*_ → 0. Second, we prove a property akin to ergodicity, the local equivalence between temporally and thermally averaged ensembles ∥*ρ*_*∞*_−*g*_*β*_∥_*A*_ → 0. Once these two statements are proven, one obtains the desired result via a triangle inequality.

A sufficient condition for quantum equilibration is well known when assuming nondegenerate spectral gaps: the vanishing of the IPR^24^, leading to

$${\mathbb{E}}_{\psi} \,{\mathbb{E}}_{t}\left[{\parallel \psi (t)-{\rho }_{\infty }\parallel }_{A}\right]\le {2}^{{N}_{A}}{\mathbb{E}}_{\psi} {\left[{\sum }_{j=1}^{{2}^{N}}{| \langle {E}_{j}| \psi \rangle | }^{4}\right]}^{1/2},$$

which gives the last term in Eq. (8) upon using property (b) of an EDGE.

To prove the equivalence of ensembles, we utilize a modified version of a seminal result, called the weak eigenstate thermalization hypothesis (weak ETH)^29,43–45^.

**Proposition 1** (A corollary of the weak ETH). Consider any translation-invariant, geometrically local Hamiltonian for *N* qubits in a *D*-dimensional periodic lattice, with an eigenbasis ${\{\vert {E}_{j}\rangle \}}_{j}$ which is common to the translation operator. Assume that the Gibbs state *g*_*β*_ has a finite correlation length and consider a geometrically local region *A* with a constant number of qubits. Then,

9 $${\sum }_{j=1}^{{2}^{N}}\langle {E}_{j}| {g}_{\beta }| {E}_{j}\rangle {\left\Vert \vert {E}_{j}\rangle \langle {E}_{j}\vert -{g}_{\beta }\right\Vert }_{A}\le O({N}^{-\gamma }),$$

for any *γ* < 1/(*D* + 1) at any temperature and for any *γ* < 1/2 at sufficiently high temperature.

The proof of Proposition 1, which leverages results from refs. ^45,48,49^, is available in the Supplementary Note. By applying this result to *ρ* = *g*_*β*_, we establish the equivalence between the thermal and temporal ensembles with bounded error:

$${\mathbb{E}}_{\psi} {\parallel {\rho }_{\infty }-{g}_{\beta }\parallel }_{A}={\mathbb{E}}_{\psi} {\left\Vert {\sum}_{j}{| \langle {E}_{j}| \psi \rangle | }^{2}(\vert {E}_{j}\rangle \langle {E}_{j}\vert -{g}_{\beta })\right\Vert }_{A}\\ \le {\sum}_{j}{\mathbb{E}}_{\psi} \left[\langle {E}_{j}| \psi| {E}_{j}\rangle \right]{\left\Vert \vert {E}_{j}\rangle \langle {E}_{j}\vert -{g}_{\beta }\right\Vert }_{A}\\ \le {\sum}_{j}{p}_{j}{\left\Vert \vert {E}_{j}\rangle \langle {E}_{j}\vert -{g}_{\beta }\right\Vert }_{A}+2{\varepsilon }_{{{{\rm{GE}}}}}(N),$$

where we used ${\rho }_{\infty }={\sum }_{j}{| \langle {E}_{j}| \psi \rangle | }^{2}\vert {E}_{j}\rangle \langle {E}_{j}\vert$ for a given $\left\vert \psi \right\rangle$ (owing to the nondegenerate spectral gap condition) in the first line, the triangle inequality in the second line, and the substitution of ${\mathbb{E}}_{\psi} [\psi ]\approx {g}_{\beta }$ up to a bounded error 2^−*N*^*ε*_GE_(*N*) in the third line. This completes the proof.

In the Supplementary Note we present generalizations of the EDGE theorem to systems with additional conserved quantities, generalized Gibbs ensembles^50^ and finite time^51^.

### EDGEs of shallow complexity at finite temperature

While we have proven the thermalization of typical states in EDGEs, it remains to show explicit examples or at least the existence of these ensembles under physical conditions. At infinite temperature, they can be easily constructed—the random product-state ensemble constitutes an EDGE, regardless of the Hamiltonian. At finite temperature, however, such ensembles necessarily depend on the details of the Hamiltonian. In what follows, we prove that EDGEs of shallow complexity always exist at high temperature. We establish this in two steps. First, we show that, at high enough temperature, any maximally entropic ensemble (P3) of any complexity ${{{\mathcal{C}}}}$ (including the most stringent case ${{{\mathcal{C}}}}=0$) are Gibbs ensembles. Second, we show that whenever ${{{\mathcal{C}}}}(N)$ is shallow (P1), such an ensemble is energy dispersed.

**Lemma 1.** For any geometrically local Hamiltonian and any choice of complexity ${{{\mathcal{C}}}}(N)$, there exists a constant threshold ${\beta }_{*}^{({{{\mathcal{C}}}})}\ge {\beta }_{*}^{({{{\rm{sep}}}})}$ such that any maximally entropic ensemble ${{{{\mathcal{E}}}}}_{\beta,{{{\mathcal{C}}}}}$ with complexity ${{{\mathcal{C}}}}$ and inverse temperature $| \beta | \le {\beta }_{*}^{({{{\mathcal{C}}}})}$ averages to the Gibbs state, ${\mathbb{E}}_{\psi} [\psi ]={g}_{\beta }$. Furthermore, the threshold temperature does not increase as the complexity increases: ${\beta }_{*}^{({{{\mathcal{C}}}})}\ge {\beta }_{*}^{({{{{\mathcal{C}}}}}^{{\prime} })}$ if ${{{\mathcal{C}}}}\ge {{{{\mathcal{C}}}}}^{{\prime} }$.

The statement follows directly from a recent result from quantum information theory. Reference ^47^ proves that the Gibbs state of any local Hamiltonian below a threshold inverse temperature ${\beta }_{*}^{({{{\rm{sep}}}})} > 0$ is separable, by an explicit construction of a product-state Gibbs ensemble. Because the Gibbs state uniquely maximizes the von Neumann entropy, this ensemble is a maximally entropic ensemble with complexity ${{{\mathcal{C}}}}=0$ and thus any other maximally entropic ensemble at the same temperature also must average to the same unique Gibbs state. As the complexity constraint is relaxed away from product states, the threshold temperature cannot increase. It is not clear if it strictly decreases, i.e., if the Gibbs state at lower temperatures can be unraveled into an ensemble of low complexity states. This question deserves future investigation.

Now we turn to show the energy dispersion, for which we leverage the translation invariance.

**Theorem 3** (Bound on average IPR). In a periodic lattice of *N* qubits, consider an ensemble ${{{\mathcal{E}}}}$ of pure states with shallow complexity ${{{\mathcal{C}}}}$, whose average state $\rho={\mathbb{E}}_{\psi} [\psi ]$ satisfies (i) translation invariance, (ii) finite correlation length, and (iii) exponentially small subsystem purity $[{{{\rm{tr}}}}({\rho }_{A}^{2})\le {e}^{-{{\Omega }}({N}_{A})}]$. Then, the average IPR in an eigenbasis ${\{\left\vert j\right\rangle \}}_{j}$ of the translation operator is bounded,

10 $${\mathbb{E}}_{\psi} \left[{\sum }_{j=1}^{{2}^{N}}| \langle j| \psi \rangle {| }^{4}\right]\le O({N}^{-1+\delta })$$

for any *δ* > 0.

We prove in the Supplementary Note that the three properties required on *ρ* in Theorem 3 are satisfied by the Gibbs state at high temperatures ∣*β*∣ < β_*_, so taking $\vert j\rangle=\vert {E}_{j}\rangle$, and combining Theorem 3, Lemma 1 and the EDGE Theorem, we obtain our main result, Theorem 1. While the detailed proof of Theorem 3 is presented in the Supplementary Note, below we illustrate the key intuition by proving a simplified version in 1D.

*Proposition* 2. The average IPR on any eigenbasis $\left\vert j\right\rangle$ of the translation operator over the uniform ensemble of computational basis states $\left\vert b\right\rangle$ with *b* ∈ {0, 1}^*N*^ is upper bounded

11 $${\mathbb{E}}_{b}\left[{\sum }_{j=1}^{{2}^{N}}| \langle j| b\rangle {| }^{4}\right]\le \frac{1}{N}+O({2}^{-N/2}).$$

Note that Proposition 2, combined with the EDGE Theorem, already proves a novel result: that a random computational basis state will typically thermalize to infinite temperature for any translation-invariant Hamiltonian with nondegenerate spectral gaps in 1D.

**Fig. 3:**
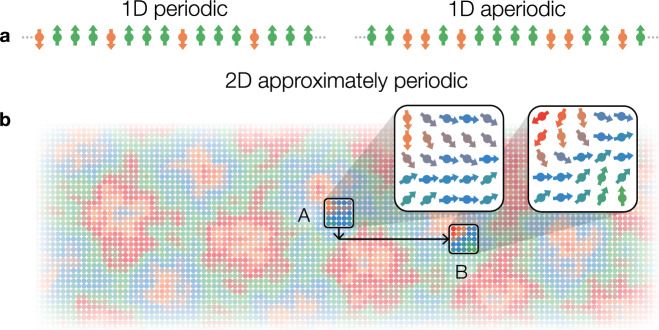
Schematic illustrations of product states on 1D or 2D lattices. **a** Periodic (left) and aperiodic (right) computational basis states in 1D. **b** Approximately periodic product state in 2D: qubit configurations in regions A and B separated by a period are similar, giving rise to long-range correlations.

*Proof*. The central idea is to divide the bitstrings *b* ∈ {0, 1}^*N*^ that enumerate the states into two different groups and bound their contributions to the average IPR separately: periodic bitstrings, which are invariant under some nontrivial translation on the circle, and aperiodic ones, which are not equal to any of their nontrivial translations (see Fig. 3a). The former group constitutes a vanishingly small fraction among all 2^*N*^ possibilities, hence its contribution becomes small. Specifically, since the period cannot be longer than *N*/2, there are at most *O*(2^*N*/2^) distinct periodic bitstrings, leading to the second term in Eq. (11). The second group contains the majority of the bitstrings, aperiodic ones, but their overlaps with the translation-invariant eigenstates are necessarily small. Specifically, for *N* distinct bitstrings *b*_1_, *b*_2_, …, *b*_*N*_ which are translations of each other, their overlaps with an eigenstate of the translation operator must be the same up to a global phase, resulting in a bound ∣〈*j*∣*b*〉∣^2^ ≤ 1/*N*. This leads to the first term in Eq. (11), completing the proof.

Theorem 3 is proven by extending the previous argument to arbitrary shallow-complexity states defined on 1D or higher-dimensional lattices. This generalization involves solving two nontrivial complications. First, unlike the bitstring case, one cannot sharply differentiate periodic versus aperiodic states because generic states have a nonzero overlap with their translations. This is overcome by introducing *approximately periodic states*, which are those states whose overlap with some translation is above a certain threshold (Fig. 3b). Second, in general, bounding the contribution to the IPR from approximately periodic states requires going beyond simple counting. For this, we utilize property (ii), the finite correlation length of *ρ*. Intuitively, approximately periodic states cannot be weighted by high probabilities in the ensemble because otherwise they would induce long-range correlations in *ρ* (see Fig. 3). This argument implicitly requires that the local regions *A* and *B* have a sufficient amount of entropy to support the correlation, hence property (iii).

### Generalized Gibbs state and finite-time thermalization

So far, we have focused on thermalization to the canonical ensemble, where the Gibbs state is specified by only one conserved quantity: energy. In the Supplementary Note we present generalizations of our results for thermalization to generalized Gibbs states ${g}_{\{{\lambda }_{i}\}}\propto \exp (-{\sum }_{i}{\lambda }_{i}{Q}_{i})$ with a constant number of conserved quantities [*Q*_*i*_, *H*] = 0. An analogous statement to Theorem 1 holds if ${\max }_{i}| {\lambda }_{i}| \le {\lambda }_{*}$ for a certain threshold λ_*_, which is independent of the system size (a generalization of *high temperature*). Our result is applicable also to certain integrable systems^52^, as long as the initial state is drawn from a generalized maximally entropic ensemble in which only a finite number of generalized chemical potentials *λ*_*i*_ are nonzero.

Furthermore, while so far we have only discussed thermalization in the infinite-time limit, our work can be improved to provide finite-time statements leveraging the results from ref. ^51^. In the Supplementary Note, we present a bound on the thermalization time in terms of the inverse of the minimal *gap of gaps*, which is expected to be exponentially small in the system size. Such exponential dependence might be unavoidable in our setting, as recent developments identify translation-invariant systems which can thermalize only after exponentially long timescales^53^. It is an important question to identify what additional physical conditions can guarantee a fast quantum thermalization in closed quantum systems.

## Discussion

It has been widely expected that statistical mechanics should be understood as an emergent phenomenon out of a microscopic quantum description^2,22,54^. Our rigorous results prove this expectation in a large class of models. We stress that even at high temperatures where the Gibbs state is separable, quantum thermalization necessitates a large amount of entanglement. The system is initialized in a low-entanglement state, which eventually becomes locally indistinguishable from the volume-law entropic Gibbs state, implying that the entanglement entropy of any subsystem grows over time and saturates to a volume-law scaling.

Our Theorem 1 establishes a universal statement of quantum thermalization which is agnostic of the details of the Hamiltonian. In practice, however, one often has access to more fine-grained information, and our several other technical results might be useful. For example, in 1D systems, the Gibbs state always has a finite correlation length, so the EDGE theorem is applicable at any temperature.

Our work leaves several other questions and generalizations open. First, in going beyond translation-invariant systems, one open question is whether one can prove a version of the weak ETH in the presence of weak disorder. However, strongly disordered systems in 1D systems are largely discussed to exhibit many-body localization^3,4,36^, which, if it exists, would constitute a robust violation of quantum thermalization.

Second, it would be desirable to prove that the nondegenerate gap condition is generically satisfied. Unlike the translation invariance and the high temperature conditions, the nondegenerate gap condition is hidden in the energy spectrum, and hence difficult to check. Nevertheless, it is expected to generically hold true, in a precise sense. Violations of the nondegenerate spectral gap condition are proven to be zero-measure for arbitrary local Hamiltonians^55^, implying that those are rare, fine-tuned exceptions. For translation-invariant Hamiltonians, the same is conjectured to hold for every system size *N*^31^, but has not yet been rigorously proven. A breakdown of this conjuncture would have counterintuitive consequences: that for some values of *N*, every translation-invariant Hamiltonian necessarily has at least one gap degeneracy and consequently hidden noninteracting degrees of freedom. This seems unlikely, and has already been ruled out by explicit numerical tests for relatively small *N*^27^.

Third, it is important to prove thermalization at lower temperatures. Away from critical points, even at low temperature, the thermal system may be described by (a mixture of a few) Gibbs state(s) with finite correlation length. A nontrivial task is to show whether or not such Gibbs states can be unraveled into an ensemble of pure shallow-complexity states. This structural question is very interesting on its own.

More broadly, one should investigate the thermalization of more general physical systems beyond qubits on lattices, such as bosonic particles with unbounded local Hilbert spaces, those possessing non-abelian symmetries (e.g., arising from spatial inversion), and quantum field theories, with the ultimate aim of constructing a universal framework explaining the emergence of statistical mechanics in quantum systems.

## Supplementary information

Supplementary Information

Transparent Peer Review file
