# Supplementary material for: Quantum thermalization must occur in translation-invariant systems at high temperature
Source: Nat Commun. 2025 Dec 16;17:75. doi: 10.1038/s41467-025-66777-7 (PMC12770369; doi:10.1038/s41467-025-66777-7)
Supplement: Supplementary file 1 — Supplementary Information [file 41467_2025_66777_MOESM1_ESM.pdf]

Supplementary Note: Quantum thermalization must occur in translation-invariant systems at high temperature

Saúl Pilatowsky-Cameo[1,*] and Soonwon Choi[1,†]

[1] *Center for Theoretical Physics, Massachusetts Institute of Technology, Cambridge, MA 02139, USA*

In this Supplementary Note, we provide detailed information, complete rigorous proofs, and generalizations of our results. In Section I, we introduce our notations, the physical setup under consideration, a detailed discussion of the nondegenerate spectral gap condition, and some useful properties of Gibbs states and shallow-complexity states to be used in later sections. Sections II, III, and IV are dedicated to formally present and prove our technical theorems outlined in the main text. Specifically, Section II contains the detailed proof of our EDGE Theorem, Section III presents the detailed definition of maximally entropic ensembles and their connection to Gibbs ensembles at high temperature, and Section IV contains the detailed proof of our bound on the average IPR, Theorem 3. Finally, in Section V we generalize our results to generalized Gibbs states in the presence of additional conserved quantities.

CONTENTS

I. Preliminaries 2
- A. Setting and notations 2
  - 1. Asymptotic notation 2
  - 2. Lattice 2
  - 3. Hilbert space, observables and Hamiltonian 2
  - 4. Local trace norm 2
- B. Nondegenerate spectral gaps 3
  - 1. Physical meaning of the nondegenerate spectral gap condition 3
  - 2. Genericity of nondegenerate spectral gaps 4
- C. Properties of Gibbs states 4
  - 1. Maximum entropy principle 4
  - 2. Finite correlation length 5
  - 3. Separability at high temperature 5
  - 4. Small subsystem purity 6
- D. Depth complexity and shallow-complexity states 6

II. The EDGE theorem 7
- A. Equivalence of the temporal and thermal ensembles 8
- B. Quantum equilibration 10
- C. Finite-time thermalization 10

III. Maximally entropic ensembles of shallow-complexity states at finite temperature 11
- A. Maximally entropic ensembles are Gibbs ensembles at high temperature 12
- B. Non-MEE ensembles of product states 12

IV. Bound on average IPR 13
- A. Aperiodic states have low IPR 13
- B. Approximately periodic states are atypical 14

V. Thermalization to generalized Gibbs states 16
- A. Properties of the generalized Gibbs state 17
- B. Generalized EDGE theorem 18
- C. Shallow-complexity G-MEEs are G-EDGEs at low generalized chemical potential 19

---

* saulpila@mit.edu
† soonwon@mit.edu

I. PRELIMINARIES

A. Setting and notations

We begin by introducing our physical setup and general notations.

1. Asymptotic notation

We make use of the big-O and related asymptotic notations. When we write $f(N) \leq O(g(N))$, we mean that there exist constants $C > 0$ and $N_* > 0$ such that $f(N) \leq Cg(N)$ for all $N \geq N_*$. We sometimes also write $f(N) \leq e^{O(g(N))}$, which means $f(N) \leq e^{Cg(N)}$ for all $N \geq N_*$. For lower bounds, we write $f(N) \geq \Omega(g(N))$ which means $f(N) \geq Cg(N)$ for all $N \geq N_*$ and $f(N) \leq e^{-\Omega(g(N))}$ means $f(N) \leq e^{-Cg(N)}$ for all $N \geq N_*$. Finally, $f(N) = \Theta(g(N))$ means that both the upper bound $f(N) \leq O(g(N))$ and lower bound $f(N) \geq \Omega(g(N))$ are satisfied.

We remark that these relations are typically expressed using equality rather than inequality signs. However, we find that the inequalities provide additional clarity.

2. Lattice

We consider systems defined over a D-dimensional lattice $\Lambda = \{0, 1, 2, \dots, L-1\}^D$, consisting of $N = L^D$ sites. As it will become clear, the specific geometric properties such as the aspect ratios of the lattice are not important for any of our results. We will specialize to periodic boundary conditions, so we will think of Λ as a torus. Given a point $\boldsymbol{x} \in \Lambda$, with coordinates $\boldsymbol{x} = (x_1, \dots, x_D)$, we measure its magnitude via the Manhattan norm,

$$\|\boldsymbol{x}\|_1 = \sum_{j=1}^{D} \min\{x_j, L - x_j\}. \tag{S1}$$

A pair of positions in the lattice can be added or subtracted in the conventional way, i.e. with the entrywise modular addition and subtraction. The distance between two sites $\boldsymbol{x}, \boldsymbol{y} \in \Lambda$ is given by $\|\boldsymbol{x} - \boldsymbol{y}\|_1$.

We will study small regions (subsets) of the lattice, in the following sense.

Definition S1 (Geometrically local region)**.** We say a region $A \subseteq \Lambda$ is *geometrically local* if its diameter is bounded by a constant

$$\max_{\boldsymbol{x},\boldsymbol{y} \in A} \|\boldsymbol{x} - \boldsymbol{y}\|_1 \leq O(1). \tag{S2}$$

3. Hilbert space, observables and Hamiltonian

On each lattice site, we place a single qubit, i.e. a spin-1/2 particle, with local Hilbert space $\mathcal{H}_{\boldsymbol{x}} = \mathbb{C}^2$. The global Hilbert space is $\mathcal{H} = \bigotimes_{\boldsymbol{x} \in \Lambda} \mathcal{H}_{\boldsymbol{x}}$. We say an operator O on $\mathcal{H}$ acts on a region $A \subseteq \Lambda$ (or is supported on A) if there is an operator O_A acting on $\mathcal{H} = \bigotimes_{\boldsymbol{a} \in A} \mathcal{H}_{\boldsymbol{a}}$ such that $O = O_A \otimes \bigotimes_{\boldsymbol{x} \notin A} \mathbb{1}_{\boldsymbol{x}}$. Henceforth, we abuse notation and not distinguish between O_A and O whenever the latter is supported in A.

For a $\boldsymbol{z} \in \Lambda$, the operator $\mathbb{T}^{\boldsymbol{z}}$ is the unitary translation operator, which maps a product state $|\psi\rangle = \bigotimes_{\boldsymbol{x} \in \Lambda} |\psi_{\boldsymbol{x}}\rangle$ to $\mathbb{T}^{\boldsymbol{z}} |\psi\rangle = \bigotimes_{\boldsymbol{x} \in \Lambda} |\psi_{\boldsymbol{x}-\boldsymbol{z} \mod L}\rangle$. We study geometrically k-local, translation-invariant (TI) Hamiltonians H, which means that $H = \sum_i h_i$, where each h_i acts on a geometrically local region containing at most k qubits and has bounded operator norm, and $H = \mathbb{T}^{\boldsymbol{z}} H \mathbb{T}^{-\boldsymbol{z}}$ for any $\boldsymbol{z} \in \Lambda$. We will often just say H is local, when the number k is not important.

4. Local trace norm

We measure the local indistinguishability of two states with the following notion.

Definition S2 (Local trace norm)**.** Given an operator W, its trace norm $\|W\|$ is equal to the sum of the singular values of W. Given a region $A \subseteq \Lambda$ with complement $\bar{A}$, we denote $\|W\|_A = \|\mathrm{tr}_{\bar{A}}(W)\|$.

From the properties of the trace norm, one can see that

$$\|\rho - \sigma\|_A = \max_{\|O_A\|_{\rm op} \leq 1} |\mathrm{tr}(\rho O_A) - \mathrm{tr}(\sigma O_A)|, \tag{S3}$$

where O_A runs over all observables supported on A with operator norm $\|\cdot\|_{\rm op}$ bounded by 1. Technically, $\|\cdot\|_A$ is not a norm, but only a seminorm as $\|\rho - \sigma\|_A = 0$ implies only that the states ρ and σ are locally indistinguishable $\mathrm{tr}_{\bar{A}}(\rho) = \mathrm{tr}_{\bar{A}}(\sigma)$, meaning that they cannot be discriminated using observables supported on region A.

B. Nondegenerate spectral gaps

One of the most important, nontrivial properties that we demand from the spectrum of Hamiltonians is the *nondegenerate spectral gaps* condition.

Definition S3 (Nondegenerate spectral gaps)**.** A Hamiltonian has *nondegenerate spectral gaps* if its spectrum $\{E_j\}_j$ has no degeneracies and no gap degeneracies, meaning that

$$E_i + E_j = E_m + E_l \iff (i,j) = (m,l) \text{ or } (i,j) = (l,m). \tag{S4}$$

This condition is also called the second no-resonance condition [1].

1. *Physical meaning of the nondegenerate spectral gap condition*

The nondegenerate gap condition has physical interpretation: it rules out the existence of hidden noninteracting degrees of freedom, as follows.

Proposition S1 (Gap degeneracies arise because of hidden noninteracting qubits)**.** *If a Hamiltonian H violates the nondegenerate gap condition, then there exists a hidden pair of noninteracting qubits, in the following sense. One can find a four-dimensional subspace $\widetilde{\mathcal{H}} \cong \mathbb{C}^2 \otimes \mathbb{C}^2 \subseteq \mathcal{H}$ and its orthogonal complement $\widetilde{\mathcal{H}}_\perp$ such that the Hamiltonian decomposes into $H = \widetilde{H} + \widetilde{H}_\perp$, where (i) $\widetilde{H}$ acts only within $\widetilde{\mathcal{H}}$, (ii) $\widetilde{H}_\perp$ acts only within $\widetilde{\mathcal{H}}_\perp$, and (iii) $\widetilde{H}$ describes two noninteracting qubits:*

$$\widetilde{H} = a\,\widetilde{Z}_1 + b\,\widetilde{Z}_2 + c. \tag{S5}$$

Here, a, b, c are constants and $\widetilde{Z}_i$ are Pauli-Z operators acting on one of the two tensor factors which compose $\widetilde{\mathcal{H}}$.

The significance of Proposition S1 comes from the fact that $\widetilde{H}$ lacks an interaction term such as $\widetilde{Z}_1\widetilde{Z}_2$. We note that the two qubits in $\widetilde{\mathcal{H}}$ are not necessarily physical qubits, and in general $\widetilde{Z}_1$ and $\widetilde{Z}_2$ might be highly nonlocal operators in the lattice. This phenomenon is conceptually similar to the hidden noninteracting degrees of freedom that arise in free-fermionic spin-chains.

Proof. Let $\widetilde{H}$ be a subspace spanned any four different energy eigenstates $|E_i\rangle, |E_j\rangle, |E_m\rangle, |E_l\rangle$, i.e. with all indices different. We identify these states with the canonical basis of $\mathbb{C}^2 \otimes \mathbb{C}^2$, as follows

$$|\widetilde{00}\rangle = |E_m\rangle, \qquad |\widetilde{01}\rangle = |E_i\rangle, \qquad |\widetilde{10}\rangle = |E_j\rangle, \qquad |\widetilde{11}\rangle = |E_l\rangle. \tag{S6}$$

We add the symbol $\widetilde{\cdots}$ to emphasize that these are not physical spin states. We consider the Pauli operators

$$\widetilde{Z}_1 = |\widetilde{00}\rangle\langle\widetilde{00}| + |\widetilde{01}\rangle\langle\widetilde{01}| - |\widetilde{10}\rangle\langle\widetilde{10}| - |\widetilde{11}\rangle\langle\widetilde{11}| \qquad \text{and} \qquad \widetilde{Z}_2 = |\widetilde{00}\rangle\langle\widetilde{00}| - |\widetilde{01}\rangle\langle\widetilde{01}| + |\widetilde{10}\rangle\langle\widetilde{10}| - |\widetilde{11}\rangle\langle\widetilde{11}|. \tag{S7}$$

By substituting Eq. (S7) into Eq. (S5) and equating the result with

$$H|_{\widetilde{\mathcal{H}}} = E_m\,|\widetilde{00}\rangle\langle\widetilde{00}| + E_i\,|\widetilde{01}\rangle\langle\widetilde{01}| + E_j\,|\widetilde{10}\rangle\langle\widetilde{10}| + E_l\,|\widetilde{11}\rangle\langle\widetilde{11}|, \tag{S8}$$

we obtain the following set of equations

$$E_m = a + b + c, \qquad E_i = a - b + c, \qquad E_j = -a + b + c, \qquad E_l = -a - b + c, \tag{S9}$$

which have a solution if and only if $E_i + E_j = E_m + E_l$, in which case $a = \frac{1}{2}(E_j - E_m)$, $b = \frac{1}{2}(E_j - E_l)$, and $c = \frac{1}{2}(E_l + E_m)$. Because we are assuming all indices different, the equation $E_i + E_j = E_m + E_l$ amounts to a violation of the nondegenerate spectral gap condition, which is thus equivalent to the Hamiltonian $H|_{\widetilde{\mathcal{H}}}$ having the form in Eq. (S5). $\square$

2. Genericity of nondegenerate spectral gaps

The nondegenerate spectral gap condition is believed to hold for generic Hamiltonians. It has been shown that all Hamiltonians violating this condition form a zero measure set in the space of k-local Hamiltonians on N qubits for all N [2]. Even within the space of *translation-invariant* Hamiltonians on N qubits, it is conjectured that all exceptions form a zero measure set. The latter space can be parametrized, for example, as

$$H = \sum_{\boldsymbol{n}\in\Lambda} \mathbb{T}^{\boldsymbol{n}} \sum_{P} J_P P \mathbb{T}^{-\boldsymbol{n}}, \tag{S10}$$

where P runs over all Pauli strings which act on some fixed geometrically local region of k qubits, and the coefficients are $J_P \in [-1, 1]$.

Conjecture S1 (Conjecture 2 of Ref. [3]: almost all k-local TI Hamiltonians have nondegenerate gaps)**.** *For a fixed $k \geq 2, N$, and D, almost all (geometrically) local TI Hamiltonians on the D-dimensional periodic lattice with N qubits have nondegenerate spectral gaps, with respect to the ensemble produced by taking the coefficients J_P in Eq.* (S10) *to be uniformly distributed in* $[-1, 1]$.

Although proving conjecture S1 remains an open problem, Ref. [3] has made some progress by showing the following:

Theorem S1 (Lemma 8 of [3])**.** *For a fixed $k \geq 2, N$, and D, one (and only one) of the following statements holds:*

1. *Almost all k-local TI Hamiltonians on the D-dimensional periodic lattice with N qubits have nondegenerate spectral gaps.*

2. *No k-local TI Hamiltonian on the D-dimensional periodic lattice with N qubits has nondegenerate spectral gaps.*

Conjecture S1 asserts that case 1 in the above theorem is the true statement. Thus, proving the conjecture only requires showing that case 2 does not occur, which reduces to finding a single example of a k-local TI system with nondegenerate spectral gaps. This has been done numerically [4] for accessible system sizes. For all systems sizes N checked so far, case 1 holds as expected. However, for arbitrary system sizes, it appears difficult to attain analytical understanding of the spectrum of a Hamiltonian with nondegenerate spectral gaps. This is an interesting and important problem that we do not address in this work.

C. Properties of Gibbs states

The Gibbs state of H at inverse temperature β is denoted by

$$g_\beta = \frac{e^{-\beta H}}{\mathrm{tr}(e^{-\beta H})}. \tag{S11}$$

As mentioned in the main text, we always use units where the Boltzmann constant is $k_B = 1$. Without loss of generality, we will only consider nonnegative temperatures $\beta \geq 0$ (a change on the overall sign of the Hamiltonian always ensures this). Below, we introduce several known properties satisfied by Gibbs states under certain conditions that we use throughout our work.

1. Maximum entropy principle

The defining property of the Gibbs state is that it uniquely maximizes the von Neumann entropy $S(\rho) = -\mathrm{tr}(\rho \log \rho)$ among all states sharing the same energy expectation value $E_\beta = \mathrm{tr}(g_\beta H)$, i.e.,

$$g_\beta = \underset{\rho:\mathrm{tr}(\rho H)=E_\beta}{\mathrm{argmax}} \; S(\rho). \tag{S12}$$

This can be shown by minimizing the relative entropy $D(\rho||g_\beta) = -S(\rho) + \beta\,\mathrm{tr}(H\rho) + \log\big(\mathrm{tr}\big(e^{-\beta H}\big)\big)$.

2. *Finite correlation length*

The correlation length of a state denotes the effective distance over which correlations, whether classical or quantum, can persist. In many systems, particularly those at high temperatures, the Gibbs state exhibits a finite correlation length, defined as follows.

Definition S4 (Finite correlation length)**.** Let ρ be a state. We define the *correlations between A and B* by

$$\mathrm{Corr}_\rho(A,B) := \max_{O_A,O_B} \frac{|\mathrm{tr}(\rho O_A \otimes O_B) - \mathrm{tr}(\rho O_A)\,\mathrm{tr}(\rho O_B)|}{\|O_A\|_{\mathrm{op}}\|O_B\|_{\mathrm{op}}} \tag{S13}$$

where O_A (O_B) runs over all operators with support contained in region A (B). We say ρ has *finite correlation length* $\xi > 0$ if for any regions A and B,

$$\mathrm{Corr}_\rho(A,B) \le f_{\mathrm{corr}}(a) e^{-d(A,B)/\xi},$$

where $f_{\mathrm{corr}}(a)$ is a fixed polynomial function, evaluated at $a = \min\{|\partial A|, |\partial B|\}$, with $|\partial A|, (|\partial B|)$ the size of the boundary of A (B), i.e., the number of pairs of contiguous sites such that one site is inside the region and the other outside. The distance between A and B is given by

$$d(A,B) = \min\{\|\boldsymbol{x}-\boldsymbol{y}\|_1 \,|\, \boldsymbol{x} \in A, \boldsymbol{y} \in B\}.$$

In one-dimensional systems, Gibbs states of TI local Hamiltonians have finite correlation length at nonzero temperatures, with $f_{\mathrm{corr}}(a) = 1$ independent of a [5, 6]. For higher dimensions, Gibbs states are guaranteed to have finite correlation length above a certain temperature, as shown by the following result.

Lemma S1 (Theorem 2 of [7]: High-temperature Gibbs states have finite correlation length)**.** *Let g_β be the thermal state at inverse temperature β of a Hamiltonian defined over a collection of spins. There exist an inverse temperature $\beta_*^{(\mathrm{corr})} > 0$ such that for every $\beta < \beta_*^{(\mathrm{corr})}$, there is a constant ξ' such that the Gibbs state g_β satisfies $\mathrm{Corr}_\rho(A,B) \le 4ac\, e^{-d_H(A,B)/\xi'}$ for any regions A, B with $d_H(A,B) \ge L_0(a) := \xi'|\log(ac)|$, where $a = \min\{|\partial^{(H)}A|, |\partial^{(H)}B|\}$ and $c = (\log(3)(1-e^{-1/\xi'}))^{-1}$.*

In Lemma S1, the geometry is determined by the interaction terms of the general Hamiltonian, which subsequently determines the distance between regions $d_H(A,B)$ and their boundaries $\partial^{(H)}A, \partial^{(H)}B$. Specifically, Lemma S1 considers the *interaction hypergraph* of H, whose hyperedges are the supports of each of the interactions terms which make up H. The distance between two points in the interaction graph is the shortest path of hyperedges that connect them, and the boundary of a region consists of all hyperedges that contain a site inside the region and a site outside.

In our setting, Lemma S1 states that the Gibbs state has finite correlation length for $\beta < \beta_*^{(\mathrm{corr})}$, given by

$$\beta_*^{(\mathrm{corr})} = \frac{1}{2\max_i \|h_i\|_{\mathrm{op}}} \log\Big(1 + \sqrt{1 + 4/\alpha}\Big), \tag{S14}$$

with $\alpha \ge ((2R+1)^D - 1)e$ [7], where R is the side length of a hypercubical region large enough to fit the (translated) support of any of the interaction terms h_i appearing in the Hamiltonian. The distance in the interaction hypergraph is related to the distance on the lattice by $d_H(A,B) \ge d(A,B)/k$. The extra factor k arises because the support of h_i can have at most diameter k in the lattice. Similarly $|\partial^{(H)}A| \le b_1|\partial A|$, (with b_1 an unimportant constant which depends on k and D). Because of the relations above, as long as $\beta < \beta_*^{(\mathrm{corr})}$, the Gibbs state has finite correlation length $\xi \le \xi'/(Dk)$, with $f_{\mathrm{corr}}(a) = b_1 4c^2a^2$. The extra factor (ca) in f_{corr} guarantees that the correlations are bounded for any distance $d(A,B)$, as we demand in Definition S4, even if this distance is smaller than L_0.

3. *Separability at high temperature*

Recently, Ref. [8] showed that in any local qubit Hamiltonian, there is a constant temperature above which the Gibbs state is separable: it can be written exactly as a mixture of product states.

Lemma S2 (Theorem 1.5 of [8]: High-temperature Gibbs states are separable)**.** *Consider a Hamiltonian consisting of Pauli-string interactions on qubits*[1]*, where each qubit appears in at most $\mathfrak{d}$ terms and each term contains at most $\mathfrak{K}$*

[1] Any local Hamiltonian can be written in terms of Pauli-string interactions.

qubits. For $|\beta| < \beta_*^{(\mathrm{sep})} := 1/(\gamma\mathfrak{d}\mathfrak{K}^2)$ *with a fixed universal constant* γ*, the corresponding Gibbs state can be expressed as a mixture*

$$g_\beta = \sum_{|\psi\rangle\in\mathcal{S}^{\otimes N}} p_\psi \, |\psi\rangle\!\langle\psi| \, , \tag{S15}$$

of product states in $\mathcal{S}^{\otimes N}$*, where* $\mathcal{S} = \{|0\rangle, |1\rangle, |+\rangle, |-\rangle, |+i\rangle, |-i\rangle\}$*.*

The states in $\mathcal{S}^{\otimes N}$ are product stabilizer states, i.e., $|\pm\rangle$, $|\pm i\rangle$ and $|0/1\rangle$ are the eigenstates of Pauli X, Y, and Z, respectively. The local terms of the Hamiltonian in Lemma S2 are assumed to have operator norm bounded by unity, which in our setting can always be achieved by dividing H by $\max_i \|h_i\| \le O(1)$, thus effectively rescaling $\beta_*^{(\mathrm{sep})} = 1/(\max_i \|h_i\|\gamma\mathfrak{d}\mathfrak{K}^2)$. The proof of Lemma S2 is constructive: Ref. [8] provides an explicit algorithm to efficiently sample from the distribution p_ψ.

4. Small subsystem purity

We will be studying the reduced Gibbs state $g_{\beta,A} = \mathrm{tr}_{\bar{A}}(g_\beta)$ within a region A. Intuitively, we expect the entropy of such state to be extensive, i.e., proportional to the number of qubits N_A in A. Below we prove this intuition at high temperature, phrased in terms of the subsystem purity being exponentially small in N_A.

Proposition S2. *Let* H *be a local Hamiltonian defined over a* D*-dimensional periodic lattice and* A *a region with* N_A *qubits. For* $\beta \le \beta_*^{(\mathrm{sep})}$*, the subsystem purity of the Gibbs state is bounded as*

$$\mathrm{tr}\big((g_{\beta,A})^2\big) \le e^{-\Omega(N_A)}. \tag{S16}$$

Proof. We utilize the decomposition of the Gibbs state into stabilizer product states of Ref. [8], given by Lemma S2. We leverage following property of this probability distribution:

Lemma S3. *Consider the distribution on* $\mathcal{S}^{\otimes N}$ *given by Lemma S2. There exists a positive constant* $c > 0$ *such that, for any* $|s\rangle \in \mathcal{S}$*, any site* $\boldsymbol{j} \in \Lambda$*, and any geometrically local* $B \subseteq \Lambda \setminus \{\boldsymbol{j}\}$*, conditioned on all the sites in* B *being on an arbitrary stabilizer product state* $|\psi_B\rangle \in \mathcal{S}^{\otimes|B|}$*, the probability of* $|\psi_{\boldsymbol{j}}\rangle = |s\rangle$ *is lower bounded by* c*:*

$$\mathbb{P}\big[\,|\psi_{\boldsymbol{j}}\rangle = |s\rangle \;\big|\; |\psi_B\rangle\big] \ge c. \tag{S17}$$

Note that Eq. (S3) is stronger than just saying that the marginal probability is lower bounded $\mathbb{P}\big[\,|\psi_{\boldsymbol{j}}\rangle = |s\rangle\,\big] \ge c$. Lemma S3 states that even when knowing the state of other sites, we can still expect any of the six outcomes for site $\boldsymbol{j}$ may occur with probability at least c. Lemma S3 follows straightforwardly from analyzing the sampling algorithm of Ref. [8], where we can see that any of the six outcomes may appear with constant probability as each qubit is *pinned*. Specifically, Algorithm 6.3 of Ref. [8] outputs $\mathbb{1}/2$ as the state of the qubit with constant probability [9].

To apply Lemma S3, we write

$$\mathrm{tr}\big((g_{\beta,A})^2\big) = \sum_{|\phi\rangle,|\psi\rangle\in\mathcal{S}^{\otimes N}} p_\psi p_\phi |\langle\psi_A|\phi_A\rangle|^2 \le \sum_{|\phi\rangle\in\mathcal{S}^{\otimes N}} p_\phi \mathbb{P}_\psi[\forall \boldsymbol{j} \in A : |\psi_{\boldsymbol{j}}\rangle \ne |\phi_{\boldsymbol{j}}\rangle^\perp], \tag{S18}$$

where the probability $\mathbb{P}_\psi$ is taken with respect to the distribution p_ψ, and $|\phi_{\boldsymbol{j}}\rangle^\perp$ denotes the single-site state perpendicular to $|\phi_{\boldsymbol{j}}\rangle$, i.e., $|0\rangle^\perp = |1\rangle$, $|+\rangle^\perp = |-\rangle$, etc.

By applying the chain rule for probability, we can use Lemma S3 on each site in $A = \{\boldsymbol{a}_1, \dots, \boldsymbol{a}_{N_A}\}$, conditioning on the *previous* sites:

$$\mathbb{P}_\psi[\forall \boldsymbol{j} \in A : |\psi_{\boldsymbol{j}}\rangle \ne |\phi_{\boldsymbol{j}}\rangle^\perp] = \prod_{k=1}^{N_A} \mathbb{P}_\psi\big[\,|\psi_{\boldsymbol{a}_k}\rangle \ne |\phi_{\boldsymbol{a}_k}\rangle^\perp \,\big|\, |\,\psi_{A\setminus\{\boldsymbol{a}_1,\dots,\boldsymbol{a}_k\}}\rangle\big] \le (1-c)^{N_A}. \qquad \square$$

D. Depth complexity and shallow-complexity states

We prove thermalization on systems initially prepared on some *physical* quantum state. To precisely define what we mean by physical, we use the notion of depth complexity.

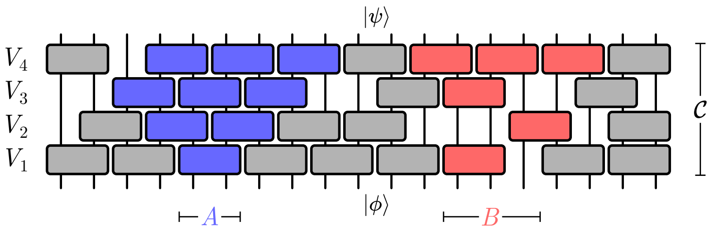

Supplementary Figure 1. State $|\psi\rangle$ with complexity $\mathcal{C} = 4$ in 1D is generated from a product state $|\phi\rangle$ by 4 circuit layers V_1-V_4. Each box represents a unitary gate. Blue and red boxes mark the light cone of two regions A and B, respectively.

Definition S5 (Shallow complexity state)**.** Given any state $|\psi\rangle$, its *(depth) complexity* $\mathcal{C}$ is the minimum number of circuit layers that are necessary to transform a product state $|\phi\rangle$ into $|\psi\rangle$,

$$|\psi\rangle = V_{\mathcal{C}} \cdots V_2 V_1 |\phi\rangle . \tag{S19}$$

where a layer is a unitary $V_j \in U(2^N)$ made up of products of geometrically local 2-qubit gates with disjoint support (Supp. Fig. 1). We say that $|\psi\rangle$ has *shallow complexity* if its depth complexity is subpolynomially growing with N, meaning that $\mathcal{C} \leq O(N^\nu)$ for any $\nu > 0$.

Note that product states themselves have shallow complexity, with trivial $\mathcal{C} = 0$, and increasing $\mathcal{C}$ increases the possible ammount of entanglement in the state. Crucially, we may upper bound the entanglement and the long-range correlations of a state via its complexity.

Lemma S4 (Bound on entanglement and correlations from depth complexity)**.** *Let $|\psi\rangle$ have depth complexity $\mathcal{C}$. Then*

(i) For any region $A \subseteq \Lambda$, the max entropy $S_0(\psi_A) = \log(\mathrm{rank}(\psi_A))$ is upper bounded by the number of sites in A which are at most a distance $\mathcal{C}$ away from the boundary, i.e. $S_0(\psi_A) \leq \log(2)|\partial_{\mathcal{C}} A|$, with $\partial_{\mathcal{C}} A = \{\boldsymbol{a} \in A \,|\, \exists \boldsymbol{b} \in \bar{A} : \|\boldsymbol{a} - \boldsymbol{b}\| \leq \mathcal{C}\}$.

(ii) The state $|\psi\rangle$ does not have correlations beyond (twice) its depth complexity. For any regions $A, B \subseteq \Lambda$ with $d(A, B) > 2\mathcal{C}$,

$$\mathrm{Corr}_\psi(A, B) = 0. \tag{S20}$$

Proof. For part *(i)* note that $\mathrm{rank}(\psi_A) \leq 2^m$, where m counts number of qubits in A which are entangled with qubits in $\bar{A}$. Because each layer is composed of local 2-qubit gates, for a qubit in A to be entangled with a qubit outside, it must be at most a distance $\mathcal{C}$ away in from the boundary, so $\mathrm{rank}(\psi_A) \leq 2^{|\partial_{\mathcal{C}} A|}$, as desired.

For part *(ii)*, note that $\langle\psi| O_A \otimes O_B |\psi\rangle = \langle\phi| U^\dagger (O_A \otimes O_B) U |\phi\rangle$, where $U = V_{\mathcal{C}} \cdots V_2 V_1$ is the circuit that maps the product state $|\phi\rangle$ into $|\psi\rangle$. If $d(A, B) \leq 2\mathcal{C}$, then the support of $U^\dagger O_A U$ does not intersect that of $U^\dagger O_A U$ —it is said that A lies outside of the *light cone* of B, as illustrated in Supp. Fig. 1— so we may factorize $\langle\phi| U^\dagger O_A \otimes O_B U |\phi\rangle = \langle\phi| U^\dagger O_A U |\phi\rangle\langle\phi| U^\dagger O_B U |\phi\rangle$, or $\langle\psi| O_A \otimes O_B |\psi\rangle = \langle\psi|O_A|\psi\rangle \, \langle\psi|O_B|\psi\rangle$. □

If the state $|\psi\rangle$ has constant complexity, Lemma S4 (i) implies an area law for the entanglement of $S(\psi_A) \leq O(|\partial A|)$.

II. THE EDGE THEOREM

We are now in position to present our first result. We consider energy dispersed Gibbs ensembles (EDGEs) (see Definition 1 from the main text) and prove our EDGE theorem:

Theorem S2 (EDGE Theorem 2, extended statement)**.** *Consider any translation invariant, geometrically local Hamiltonian for N qubits in D-dimensional lattice with nondegenerate spectral gaps. Let $\mathcal{E}_\beta$ be an EDGE and assume that the Gibbs state g_β has finite correlation length. Then, for any geometrically local region A with a constant number of qubits N_A,*

$$\mathop{\mathbb{E}}_{\psi} \mathop{\mathbb{E}}_{t} \left[\|g_\beta - \psi(t)\|_A \right] \leq O(N^{-\gamma}) + 2\varepsilon_{\mathrm{GE}}(N) + 2^{N_A} \varepsilon_{\mathrm{ED}}(N)^{1/2}, \tag{S21}$$

for any $\gamma < 1/(D+1)$. Furthermore, the statement holds for any $\gamma < 1/2$ (independent of D) as long as the temperature is above a threshold $\beta < \beta_*^{(\mathrm{con})}$ given by $(\beta_*^{(\mathrm{con})})^{-1} = 8e^3 \times \max\{N_A^2, \mathfrak{d}\mathfrak{K} \max_i \|h_i\|\}$, where, in the Hamiltonian, each qubit appears in at most $\mathfrak{d}$ terms and each term h_i contains at most $\mathfrak{K}$ qubits.

The rest of this section is devoted to proving Theorem S2. Our proof is inspired by Ref. [3], which studies 1D systems at infinite temperature. We will show that a typical state ψ in the EDGE satisfies two properties:

(a) Equivalence of ensembles between the temporal $\rho_\infty = \mathbb{E}_t[\psi(t)]$ and the thermal ensemble g_β:

$$\mathop{\mathbb{E}}_{\psi} \|\rho_\infty - g_\beta\|_A \leq O(N^{-\gamma}) + 2\varepsilon_{\mathrm{GE}}(N),$$

(b) Equilibration:

$$\mathop{\mathbb{E}}_{\psi} \mathop{\mathbb{E}}_{t}[\|\rho_\infty - \psi(t)\|_A] \leq 2^{N_A} \varepsilon_{\mathrm{ED}}(N)^{1/2}.$$

Taken together, these two properties give us the desired quantum thermalization in Eq (S21). The equivalence of ensembles will follow from property (a) of an EDGE and a variant of the weak ETH [10–13]. We will explain this first. After, we will explain how equilibration follows from property (b) of an EDGE and the seminal results on quantum equilibration from Ref. [14].

Before proceeding, let us make a comment on the error term $O(N^{-\gamma})$ in Eq. (S21), which is the finite-size error term for the equivalence of ensembles coming from the weak ETH. As we will see below, the scaling of γ depends on known concentration bounds for the expectation value of extensive observables. The regular ETH ansatz predicts a finite-size error for thermalization of $O(N^{-1})$ ($\gamma = 1$) [15, 16], and thus even the best scaling $\gamma = 1/2 - \delta$ proven in 1D and in any dimension at high temperature is not expected to be optimal. Rigorously proving this better bound under minimal assumptions is an important further direction.

A. Equivalence of the temporal and thermal ensembles

The weak ETH guarantees that with high likelihood, the expectation value of local observables over the energy eigenstates of a translation invariant system is close to the expectation at equilibrium, with probability exponentially close to 1. It was originally derived in Refs. [10, 11], and then further improved in different settings [12, 13, 17]. We present here a modified version which is favorable for our purposes.

Proposition S3 (A variant of the weak ETH). *Let H be a TI, (geometrically) local Hamiltonian on a D-dimensional periodic lattice with N qubits. Let $\rho = \sum_{j=1}^{2^N} p_j |E_j\rangle\langle E_j|$ be a diagonal state in the common eigenbasis $\{|E_j\rangle\}_j$ of H and the translation operator, that has finite correlation length. Consider a geometrically local region A. Then*

$$\mathbb{P}_{j\sim p_j}\Big[\big\| |E_j\rangle\langle E_j| - \rho \big\|_A \geq \varepsilon\Big] \leq e^{-\Omega(N^\nu \varepsilon)}, \tag{S22}$$

for $\nu = 1/(D+1)$ and $\varepsilon \geq \Omega(N^{-\gamma})$ with $\gamma < \nu$. Furthermore, if the state ρ is a Gibbs state of H, $\rho = g_\beta$ at sufficiently high temperature $\beta < \beta_*^{(\mathrm{con})}$ (defined in Theorem S2), then the statement holds for $\nu = 1/2$, regardless of D.

Proof. We consider an arbitrary observable supported on region A with operator norm $\|O_A\|_{\mathrm{op}} \leq 1$, over which we will optimize later to obtain the local trace norm. Following Refs. [11–13], we first show that

$$\mathbb{P}_{j\sim p_j}\Big[\langle E_j|O_A|E_j\rangle - \mathrm{tr}(O_A\rho) \geq \varepsilon\Big] \leq e^{-\Omega(N^\nu \varepsilon)}. \tag{S23}$$

The first step is to utilize the translation invariance of the Hamiltonian to turn the expectation values of O_A into expectation values of an extensive observable $\bar{O} = \sum_{\boldsymbol{n}\in\Lambda} \mathbb{T}^{\boldsymbol{n}} O_A \mathbb{T}^{-\boldsymbol{n}} - N\,\mathrm{tr}(\rho O_A)$, such that $\langle E_j|O_A|E_j\rangle - \mathrm{tr}(\rho O_A) = \frac{1}{N}\langle E_j|\overline{O}|E_j\rangle$. Then, for any $\tau \geq 0$,

$$\mathbb{P}_j\Big[\langle E_j|O_A|E_j\rangle - \mathrm{tr}(\rho O_A) \geq \varepsilon\Big] = \mathbb{P}_j\Big[e^{\tau\langle E_j|\overline{O}|E_j\rangle} \geq e^{N\tau\varepsilon}\Big] \tag{S24}$$

$$\leq e^{-N\tau\varepsilon} \sum_{j=1}^{2^N} p_j e^{\tau\langle E_j|\overline{O}|E_j\rangle} \tag{S25}$$

$$\leq e^{-N\tau\varepsilon} \sum_{j=1}^{2^N} p_j \langle E_j|e^{\tau\overline{O}}|E_j\rangle = e^{-N\tau\varepsilon}\,\mathrm{tr}\Big(\rho\, e^{\tau\overline{O}}\Big), \tag{S26}$$

where the second inequality follows from Jensen's inequality.

The purpose of recasting the inequality in this form is to utilize known concentration bounds for the expectation value of extensive local observables. Equation (S23) is proven for $\nu = 1/(D+1)$ in Ref. [13] using concentration bounds from Ref. [18]. Here, we prove that it further holds for $\nu = 1/2$ for any D whenever $\rho = g_\beta$ at high enough temperature, by leveraging the concentration bound of Ref. [17]. Specifically, we apply the following result:

Lemma S5 (Theorem 1 of Ref. [17]). *Let $\mathcal{F} = \sum_{|X|\leq k} f_X$ and $H = \sum_{|X|\leq k} h_X$ with f_X, h_X k-body operators with bounded operator norm $\sum_{X:X\ni \boldsymbol{x}} \|f_X\|_{\mathrm{op}}, \sum_{X:X\ni \boldsymbol{x}} \|h_X\|_{\mathrm{op}} \leq g$ for any site $\boldsymbol{x}$. Then, if the inverse temperature satisfies $\beta < \beta_c := (8e^3 gk)^{-1}$, the Gibbs state ρ satisfies the following inequality:*

$$\log \operatorname{tr}\left(e^{-\tau\mathcal{F}}\rho\right) \leq -\tau \operatorname{tr}(\mathcal{F}\rho) + \frac{\tau^2 B}{\beta_c - \beta - |\tau|}, \tag{S27}$$

for $|\tau| < \beta_c - \beta$ and $B := \sum_{|X|\leq k} \|f_X\|_{\mathrm{op}}$.

In our particular scenario, $\mathcal{F} = \sum_{\boldsymbol{n}\in\Lambda} \mathbb{T}^{\boldsymbol{n}} O_A \mathbb{T}^{-\boldsymbol{n}}$, $B = N\|O_A\|_{\mathrm{op}}$, so if we choose $\tau = N^{-1/2}$ we obtain $\tau^2 B/(\beta_c - \beta - |\tau|)) \leq O(1)$ and

$$\mathbb{P}_j\Big[\langle E_j|O_A|E_j\rangle - \operatorname{tr}(\rho O_A) \geq \varepsilon\Big] \leq e^{-N\tau\varepsilon} \operatorname{tr}\Big(\rho\, e^{\tau\overline{O}}\Big) \leq e^{-\Omega(N^{1/2}\varepsilon)}, \tag{S28}$$

which completes the proof of Eq. (S23) in the high temperature case. Note that the critical temperature has dependence on both the locality of the Hamiltonian and N_A^2 (because the terms of $\mathcal{F}$ are all the translates of O_A, which is a N_A-body operator).

Now, to finally obtain Eq. (S22), we need to maximize Eq. (S23) over O_A. Note, importantly, that we have to perform the maximization separately for each j. For this, we consider a finite set of observables and perform a union bound [12]. Specifically, let $\mathcal{N}$ be a $(\varepsilon/3)$-net of observables supported on A, which means that for any O_A with norm bounded by 1, there is some $O'_A \in \mathcal{N}$ such that $\|O_A - O'_A\|_{\mathrm{op}} \leq \varepsilon/3$. $\mathcal{N}$ can be taken to have at most $|\mathcal{N}| \leq O((1/\varepsilon)^\alpha)$ elements, where α is a constant which only depends on N_A. Then, for each j, there is a $O_A^{(j)} \in \mathcal{N}$ such that $\big\|\, |E_j\rangle\langle E_j| - \rho\,\big\|_A \leq \langle E_j|O_A^{(j)}|E_j\rangle - \operatorname{tr}(O_A^{(j)}\rho) + \frac{2}{3}\varepsilon$, and so

$$\mathbb{P}_j\Big[\big\|\, |E_j\rangle\langle E_j| - \rho\,\big\|_A \geq \varepsilon\Big] \leq \mathbb{P}_j\Big[\exists O_A^{(j)} \in \mathcal{N}\colon\ \langle E_j|O_A^{(j)}|E_j\rangle - \operatorname{tr}(O_A^{(j)}\rho) \geq \varepsilon/3\Big] \tag{S29}$$

$$\leq \sum_{O_A\in\mathcal{N}} \mathbb{P}_j\Big[\,\langle E_j|O_A|E_j\rangle - \operatorname{tr}(O_A\rho) \geq \varepsilon/3\Big] \leq O(\varepsilon^{-\alpha}) e^{-\Omega(N^\nu\varepsilon)} \leq e^{-\Omega(N^\nu\varepsilon)}, \tag{S30}$$

where in the last inequality we used that $\varepsilon \geq \Omega(N^{-\gamma})$ with $\gamma < \nu$. □

From Proposition S3 we can prove Proposition 1 of the main text, stated here in slightly more general form.

Corollary S1 (Generalization of Proposition 1). *Under the conditions of Proposition S3, for any $\gamma < \nu$,*

$$\sum_{j=1}^{2^N} p_j \big\|\, |E_j\rangle\langle E_j| - \rho\,\big\|_A \leq O(N^{-\gamma}) \tag{S31}$$

Proof. From Proposition S3 we obtain

$$\sum_{j=1}^{2^N} p_j \big\|\, |E_j\rangle\langle E_j| - \rho\,\big\|_A \leq \varepsilon + 2e^{-\Omega(N^\nu\varepsilon)}, \tag{S32}$$

and if we pick $\varepsilon = N^{-\gamma}$, we get $\varepsilon + 2e^{-\Omega(N^\nu\varepsilon)} \leq O(N^{-\gamma})$. □

Proposition S4 (Equivalence of temporal and thermal ensembles). *Under the assumptions of the EDGE Theorem S2,*

$$\mathop{\mathbb{E}}_{\psi\in\mathcal{E}_\beta} [\|\rho_\infty - g_\beta\|_A] \leq O(N^{-\gamma}) + 2\varepsilon_{\mathrm{GE}}(N), \tag{S33}$$

where $\rho_\infty = \mathbb{E}_t[\psi(t)]$.

Proof. By the no-degeneracy condition, the infinite-time-average of any state ρ_∞ is the diagonal ensemble $\rho_\infty = \sum_j |\langle\psi|E_j\rangle|^2 |E_j\rangle\langle E_j|$. Then

$$\begin{aligned}
\mathop{\mathbb{E}}_{\psi\in\mathcal{E}_\beta}[\|\rho_\infty(\psi) - g_\beta\|_A] &= \mathop{\mathbb{E}}_{\psi\in\mathcal{E}_\beta}\left[\Big\| \sum_j \big(|E_j\rangle\langle E_j| - g_\beta\big)|\langle\psi|E_j\rangle|^2 \Big\|_A\right] \\
&\le \sum_j \||E_j\rangle\langle E_j| - g_\beta\|_A \mathop{\mathbb{E}}_{\psi\in\mathcal{E}_\beta}\left[|\langle\psi|E_j\rangle|^2\right] \\
&= \sum_j \||E_j\rangle\langle E_j| - g_\beta\|_A \langle E_j| \mathop{\mathbb{E}}_{\psi\in\mathcal{E}_\beta}[\psi] |E_j\rangle \\
&\le \sum_j \||E_j\rangle\langle E_j| - g_\beta\|_A \left(\langle E_j|g_\beta|E_j\rangle + 2^{-N}\varepsilon_{\mathrm{GE}}(N)\right) \\
&\le \Big(\sum_j \||E_j\rangle\langle E_j| - g_\beta\|_A \langle E_j|g_\beta|E_j\rangle\Big) + 2\varepsilon_{\mathrm{GE}}(N) \\
&\le O(N^{-\gamma}) + 2\varepsilon_{\mathrm{GE}}(N),
\end{aligned}$$

where we used property (a) of the EDGE and Corollary S1. □

B. Quantum equilibration

Now we shift gears to prove equilibration. We utilize the following result from Ref. [14]:

Lemma S6 (Quantum equilibration, Theorem 1 of Ref. [14]). *Let H be a Hamiltonian which has nondegenerate spectral gaps. For any state ψ with time average $\rho_\infty = \mathbb{E}_t[\psi(t)]$, and subsystem A of dimension d_A*

$$\mathop{\mathbb{E}}_t[\|\rho_\infty - \rho(t)\|_A] \le d_A\sqrt{\mathrm{IPR}}, \tag{S34}$$

where $\mathrm{IPR} = \sum_j |\langle E_j|\psi\rangle|^4$ *is the inverse participation ratio of* $|\psi\rangle$.

Proposition S5 (EDGE equilibration). *Under the assumptions of the EDGE Theorem S2,*

$$\mathop{\mathbb{E}}_{\psi\in\mathcal{E}_\beta}[\mathop{\mathbb{E}}_t[\|\rho_\infty - \rho(t)\|_A]] \le 2^{N_A}\varepsilon_{\mathrm{ED}}(N)^{1/2} \tag{S35}$$

Proof. Averaging over all states in the EDGE $\mathcal{E}_\beta$, using that $\mathbb{E}[\,\cdot\,]^2 \le \mathbb{E}[(\,\cdot\,)^2]$, and property (b) in Definition 1 of the main text, we obtain

$$\mathop{\mathbb{E}}_{\psi\in\mathcal{E}_\beta}[\mathop{\mathbb{E}}_t[\|\rho_\infty - \rho(t)\|_A]] \le \sqrt{\mathop{\mathbb{E}}_{\psi\in\mathcal{E}_\beta}[\mathop{\mathbb{E}}_t[\|\rho_\infty - \rho(t)\|_A]^2]} \le 2^{N_A}\varepsilon_{\mathrm{ED}}(N)^{1/2}.$$ □

Combining Propositions S4 and S5, by summing Eq. (S33) and Eq. (S35) and applying a triangle inequality, we complete the proof of the EDGE Theorem S2.

In the next subsection, we present a generalization of the EDGE Theorem to finite time. We also present a generalization to generalized Gibbs states in Section V.

C. Finite-time thermalization

We note that the EDGE theorem, as stated, does not say anything about the time it takes for systems to thermalize. However, using known finite-time bounds on equilibration [19], we can easily reformulate the EDGE theorem to become a finite-time statement. For example, Eq. (24) of Ref. [20] states that, under the assumptions of Lemma S6,[2]

$$\mathop{\mathbb{E}}_{t\in[0,T]}[\|\rho_\infty - \psi(t)\|_A] \le d_A\sqrt{\mathrm{IPR}\times\left(1 + \frac{8N}{G_{\min}T}\right)}, \tag{S36}$$

[2] This bound can be slightly improved using [21, Eq. (A9)].

where $\mathbb{E}_{t\in[0,T]}[\,\cdot\,] = \frac{1}{T}\int_0^T \mathrm{d}t\,(\,\cdot\,)$ denotes a finite-time average and

$$G_{\min} = \min_{(l,m)\neq(i,j)\neq(m,l)} |(E_i - E_m) - (E_l - E_j)| \tag{S37}$$

is the smallest *gap of gaps*. This quantity is nonzero due to the nondegenerate gap condition, Eq. (S4). Using this result we obtain:

Theorem S3 (Finite-time EDGE). *Under the conditions of the EDGE Theorem S2,*

$$\mathop{\mathbb{E}}_{\psi\in E_\beta}\mathop{\mathbb{E}}_{t\in[0,T]}\left[\|g_\beta - \psi(t)\|_A\right] \leq O(N^{-\gamma}) + 2\varepsilon_{\mathrm{GE}}(N) + 2^{N_A}\sqrt{\varepsilon_{\mathrm{GE}}(N)\left(1+\frac{8N}{G_{\min}T}\right)}. \tag{S38}$$

Theorem S3 guarantees that for any typical state in the ensemble, thermalization is achieved when $8N/G_{\min}T \sim 1$, i.e. after time $T_{\mathrm{therm}} \sim N/G_{\min}$.

One can estimate how small $G_{\min}$ as a function of increasing system sizes with the following argument. Let us consider $M \sim 2^{4N}$ different gaps of gaps $\{\delta\Delta_1, \delta\Delta_2, \ldots \delta\Delta_M\}$. To simplify our analysis we model them as if they are independent random variables uniformly distributed within a range $[0, W]$. We can exactly compute the probability distribution for $\delta\Delta_{\min} \equiv \min\{\delta\Delta_i\}_{i=1}^M$ and evaluate the average $\overline{\delta\Delta_{\min}} = W/(M+1)$. Thus, we should expect $G_{\min} \sim 2^{-4N}$. This scaling can be numerically verified in translation-invariant local Hamiltonians (not presented here). Hence, the bound T_{therm} is expected to grow as $\sim 2^{4N}$. An exponential scaling in the bound is inevitable, as there indeed exist translation-invariant systems that can thermalize only after exponentially long times [22].

III. MAXIMALLY ENTROPIC ENSEMBLES OF SHALLOW-COMPLEXITY STATES AT FINITE TEMPERATURE

We present a more detailed definition of maximally entropic ensembles (MEEs), and prove some of their properties. Let us begin by precisely stating what we mean by complexity and temperature associated to an ensemble.

Definition S6 (Complexity of an ensemble). We say that an ensemble of pure states $\mathcal{E}_{\mathcal{C}}$ has *complexity* $\mathcal{C}(N)$ if any state ψ in the support of $\mathcal{E}_{\mathcal{C}}$ has complexity at most $\mathcal{C}(N)$, i.e. it can be prepared by a circuit of depth at most $\mathcal{C}(N)$ from a product state.

Definition S7 (Temperature of an ensemble). We say that an ensemble $\mathcal{E}_\beta$ of pure states has *temperature* $1/\beta$ if its average energy is matches with that of the thermal state

$$\mathop{\mathbb{E}}_{\psi\in\mathcal{E}_\beta}[\,\langle\psi|H|\psi\rangle] = \mathrm{tr}(Hg_\beta). \tag{S39}$$

We define MEEs, as follows.

Definition S8 (Maximally entropic ensemble). An ensemble $\mathcal{E}^*_{\beta,\mathcal{C}}$ with temperature $1/\beta$ and complexity $\mathcal{C}$ is a *maximally entropic ensemble* (MEE) if the von Neumann entropy $S(\rho) = -\,\mathrm{tr}(\rho\log(\rho))$ of its average state $\rho^*_{\beta,\mathcal{C}} = \mathbb{E}_{\psi\in\mathcal{E}^*_{\beta,\mathcal{C}}}[\psi]$ is maximal among all ensembles with the same temperature and complexity, i.e.

$$\mathcal{E}^*_{\beta,\mathcal{C}} \in \mathop{\mathrm{argmax}}_{\mathcal{E}_{\beta,\mathcal{C}}} S(\rho_{\beta,\mathcal{C}}). \tag{S40}$$

Importantly, note that MEEs are not unique. For example, all of the following ensembles form a MEE of product states (zero complexity) at infinite temperature $\mathcal{E}_{\beta=0,\mathcal{C}=0}$: the ensemble of all computational basis states, the ensemble of all stabilizer product states, and the ensemble of all product states (each qubit state Haar-distributed). This is simply because in all cases, the average state is the maximally entropic state $\mathbb{1}/2^N$. Beyond product states, the ensemble of outputs of a random unitary circuit of some fixed shallow depth is also a MEE, for the same reason. At finite, but high enough temperature $\beta \leq \beta_*^{(\mathrm{sep})}$, the ensemble of stabilizer product states $\mathcal{E}_{\mathcal{C}=0,\beta}$ given by Lemma S2 is a MEE, because its average state is the Gibbs state, which has maximal entropy at fixed energy. One can also transform a MEE into a different MEE: given any quantum circuit U with $\mathcal{C}$ layers, the ensemble $\mathcal{E}_{\mathcal{C},0} = \{U\,|\psi\rangle \mid |\psi\rangle \in \mathcal{E}_{\beta=0,\mathcal{C}=0}\}$ is a MEE at $\beta = 0$ with complexity $\mathcal{C}$. Furthermore, if the circuit leaves the Gibbs state invariant $U^\dagger g_\beta U = g_\beta$, then $\mathcal{E}_{\mathcal{C},\beta} = \{U\,|\psi\rangle \mid |\psi\rangle \in \mathcal{E}_{\beta,\mathcal{C}=0}\}$ is a MEE of complexity $\mathcal{C}$ for any $\beta \leq \beta_*^{(\mathrm{sep})}$.

Our main result, Theorem 1, guarantees the thermalization of any MEE of shallow complexity at sufficiently high temperature. This is a consequence of the EDGE theorem: below we prove that at high enough temperature any MEE is a Gibbs ensemble, and we further prove in Sec. IV that any Gibbs ensemble of shallow-complexity states is an EDGE.

A. Maximally entropic ensembles are Gibbs ensembles at high temperature

Here, we show that at high enough but finite temperatures, a MEE forms a Gibbs ensemble.

Lemma S7 (Lemma 1 restatement)**.** *For any geometrically local Hamiltonian and any choice of complexity $\mathcal{C}(N)$, there exists a constant threshold $\beta_*^{(\mathcal{C})} \geq \beta_*^{(\mathrm{sep})}$ which is independent of N such that any MEE $\mathcal{E}_{\beta,\mathcal{C}}$ with complexity $\mathcal{C}$ and inverse temperature $|\beta| \leq \beta_*^{(\mathcal{C})}$ averages to the Gibbs state, $\mathbb{E}_\psi[\psi] = g_\beta$. Furthermore, the threshold temperature does not increase as the complexity increases: $\beta_*^{(\mathcal{C})} \geq \beta_*^{(\mathcal{C}')}$ if $\mathcal{C} \geq \mathcal{C}'$.*

Proof. Let us fix the complexity $\mathcal{C}(N)$. Define $\beta_*^{(\mathcal{C})} > 0$ to be the smallest inverse temperature such that the Gibbs state at any $\beta \leq \beta_*^{(\mathcal{C})}$ is exactly decomposable as some mixture of pure states of circuit complexity at most $\mathcal{C}$. Note that, by the separability of the Gibbs state stated in Lemma S2, we know that $\beta_*^{(\mathcal{C})} \geq \beta_*^{(\mathrm{sep})} > 0$. Furthermore, from this definition we immediately get the monotonicity $\beta_*^{(\mathcal{C})} \geq \beta_*^{(\mathcal{C}')}$ if $\mathcal{C} \geq \mathcal{C}'$.

Now we prove that any MEE $\mathcal{E}_{\beta,\mathcal{C}}$ with inverse temperature $\beta \leq \beta_*^{(\mathcal{C})}$ and complexity $\mathcal{C}$ is a Gibbs ensemble. Let $\mathcal{E}^*_{\beta,\mathcal{C}} = \{(p_\psi, |\psi\rangle\langle\psi|)\}$ be an ensemble of pure states of complexity at most $\mathcal{C}$ such that $\mathbb{E}_\psi[\psi] = g_\beta$ (it exists by how we defined $\beta_*^{(\mathcal{C})}$). The ensemble $\mathcal{E}^*_{\beta,\mathcal{C}}$ is a MEE because the thermal state g_β precisely maximizes the von Neumann entropy $S(\rho) = -\operatorname{tr}(\rho \log \rho)$ with constrained energy [Eq. (S12)]. Furthermore, g_β uniquely satisfies this property, so the average state of any other MEE at the same temperature must also be the Gibbs state, regardless of the complexity. □

In Sec. IV we prove that, further assuming shallow-complexity, one can show that MEEs are EDGEs, which together with our EDGE theorem completes our proof of thermalization. Before this, we present a brief discussion about other ensembles of pure states for which the statement of thermalization is interesting, which are different from MEEs.

B. Non-MEE ensembles of product states

We briefly discuss other natural finite-temperature ensembles of pure states which are not obtained through our MEE construction. For simplicity, we restrict this discussion to product states. Given any probability density $p(\psi)$ in the manifold of product states, to restrict to finite temperature, one requires an energy constraint $\int d\psi p(\psi) \langle\psi|H|\psi\rangle = E_\beta$. When we define MEEs, we implicitly select p to be whatever distribution that maximizes the von Neumann entropy of the average state. However, one can select such p explicitly instead. Two natural choices are a *microcanonical product state ensemble* [9],

$$p_{(\mathrm{mic})}(\psi) = \begin{cases} \frac{1}{M} & \text{if} \quad |\langle\psi|H|\psi\rangle - \operatorname{tr}(g_\beta H)| \leq \Delta(N), \\ 0 & \text{otherwise.} \end{cases} \tag{S41}$$

for some $\Delta(N) = o(N)$ or a *canonical product state ensemble* via a Boltzmann factor

$$p_{(\mathrm{can})}(\psi) = \frac{1}{Z'} e^{-\beta' \langle\psi|H|\psi\rangle} \tag{S42}$$

where M and Z' are chosen to ensure normalization.

It is a natural question to ask whether these ensembles typically thermalize. We note, however, that one has to be careful in interpreting them as ensembles of statistical mechanics. One is effectively treating each state ψ as a distinct state with a well-defined energy $\langle\psi|H|\psi\rangle$, ignoring its quantum mechanical nature. Even if $p_{(\mathrm{mic})}(\psi) > 0$, the state ψ might still have overlap with energy eigenstates outside of the microcanonical window, because it is only the expectation value that is constrained. On the other hand the Boltzmann factor β' in $p_{(\mathrm{can})}$ will be in general different from the physical inverse temperature β, due to an over-counting in entropy, stemming from ignoring the partial indistinguishability of non-orthogonal states. We do not expect that these ensembles are Gibbs ensembles, in the sense of the EDGE theorem. Proving their thermalization is nevertheless an interesting question that we leave open. In this direction, Ref. [9] proved the equilibration (although not the thermalization) of typical states in the microcanonical product-state ensemble of Eq. (S41).

IV. BOUND ON AVERAGE IPR

We discussed in Sec. II that MEEs at high temperature satisfy property (a) of an EDGE, with $\varepsilon_{\rm GE}(N) = 0$. Here, we prove that, as long as the MEE has shallow complexity, it also satisfies property (b) of an EDGE with $\varepsilon_{\rm ED}(N) \le O(N^{-1+\delta})$, which completes the proof of Theorem 1.

We prove Theorem 3 of the main text:

Theorem S4 (Bound on average IPR, Theorem 3 restatement). *In a periodic lattice with N qubits, consider an ensemble $\mathcal{E}$ of pure states with shallow complexity $\mathcal{C}(N)$ whose average state $\rho = \mathbb{E}_{\psi\in\mathcal{E}}[\psi]$ satisfies the following three properties:*

(i) Translation invariance, $\forall \boldsymbol{n} \in \Lambda : \mathbb{T}^{\boldsymbol{n}} \rho \mathbb{T}^{-\boldsymbol{n}} = \rho$;

(ii) Finite correlation length (Definition S4);

(iii) Exponentially small subsystem purity, $\mathrm{tr}\big(\rho_A^2\big) \le e^{-\Omega(N_A)}$ for any hypercubical region A with N_A qubits.

Then, the average IPR in any eigenbasis $\{|j\rangle\}_j$ of the translation operator is bounded

$$\mathop{\mathbb{E}}_{\psi}\Big[\sum_{j=1}^{2^N} |\langle j|\psi\rangle|^4\Big] \le O(N^{-1+\delta}) \tag{S43}$$

for any $\delta > 0$.

Theorem S4 implies that a high-temperature Gibbs ensemble of shallow complexity states is an EDGE, taking $\rho = g_\beta$ and $|j\rangle = |E_j\rangle$. Property (i) is satisfied as long as the Hamiltonian is TI, by Lemma S1 property (ii) is satisfied if $\beta < \beta_*^{(\rm corr)}$, and by Proposition S2 property (iii) is satisfied if $\beta < \beta_*^{(\rm sep)}$.

The proof of Theorem S4 below generalizes that of Proposition 2 of the main text. The key idea to recall is that one may upper bound the average IPR by separately upper bounding (1) the overlap of the TI eigenstates with the aperiodic computational-basis states and (2) the probability of a bitstring being periodic. In generalizing this idea to arbitrary shallow complexity states, we face one immediate obstacle: For an aperiodic bitstring $b \in \{0,1\}^N$ the state $|b\rangle$ is orthogonal to all of its translates, $\langle b|\mathbb{T}^n|b\rangle = 0$ for all $0 < n < N$. We relied on this orthogonality in deducing that $|\langle b|j\rangle|^2 \le 1/N$ for aperiodic b. However, for a general state $|\psi\rangle$, it could be that $\langle\psi|\mathbb{T}^n|\psi\rangle \ne 0$ even if $|\psi\rangle = |\psi_1\rangle|\psi_2\rangle\cdots|\psi_N\rangle$ is a product state and the sequence $\{|\psi_1\rangle, |\psi_2\rangle, \dots, |\psi_N\rangle\}$ is aperiodic (e.g. $|\psi\rangle = |{+}0000\cdots\rangle$). To circumvent this problem, we establish a robust notion of *approximate* periodicity. We split the expectation value of the IPR into an average over approximately periodic states and the rest, and prove that (1) the IPR of states which are not approximately periodic is upper bounded by $\sim 1/N$ and (2) the approximately periodic states are atypical, i.e., they are associated with vanishingly small probabilities.

A. Aperiodic states have low IPR

Definition S9. (Approximately periodic state). An arbitrary state $|\psi\rangle$ for N qubits arranged on the D-dimensional lattice Λ is *r-approximately periodic* if there exists $\boldsymbol{n} \in \Lambda$ with $\boldsymbol{n} \ne \boldsymbol{0}$ such that $|\langle\psi|\mathbb{T}^{\boldsymbol{n}}|\psi\rangle|^2 \ge 2^{-r}$. The vector $\boldsymbol{n}$ is called a period of $|\psi\rangle$.

For now, r is arbitrary. We will select an appropriate scaling $r(N)$ at the end of the proof. We begin by generalizing our decomposition of the average IPR from Proposition 2 for approximately periodic states. We state the result in a general form, which might be useful outside of our current context.

Lemma S8 (Probabilistic bound on average IPR from symmetry). *Consider a set of symmetry operators represented by mutually commuting unitaries $\{U_1, \dots, U_M\}$. Let P be the set of approximately symmetric states, containing all ψ such that $\big|\langle\psi|U_n^\dagger U_m|\psi\rangle\big| \ge \varepsilon$ for some $n \ne m$. For any basis $\{|\alpha\rangle\}_\alpha$ of simultaneous eigenstates of all U_n, the average IPR over any ensemble of states $\mathcal{E}$ can be upper bounded as*

$$\mathop{\mathbb{E}}_{\psi\in\mathcal{E}}\Big[\sum_\alpha |\langle\alpha|\psi\rangle|^4\Big] \le \frac{1}{M} + \varepsilon + \mathbb{P}_{\psi\in\mathcal{E}}[\psi \in P], \tag{S44}$$

where $\mathbb{P}_{\psi\in\mathcal{E}}[\psi \in P]$ denotes the probability that a state drawn from the ensemble $\mathcal{E}$ is approximately symmetric.

Proof. To begin, we split the expectation value conditioning on P,

$$\mathop{\mathbb{E}}_{\psi\in\mathcal{E}}\left[\sum_\alpha |\langle\alpha|\psi\rangle|^4\right] = \mathop{\mathbb{E}}_{\psi\in\mathcal{E}}\Big[\sum_\alpha |\langle\alpha|\psi\rangle|^4\Big|\psi\notin P\Big]\mathbb{P}_{\psi\in\mathcal{E}}[\psi\notin P] + \mathop{\mathbb{E}}_{\psi\in\mathcal{E}}\Big[\sum_\alpha |\langle\alpha|\psi\rangle|^4\Big|\psi\in P\Big]\mathbb{P}_{\psi\in\mathcal{E}}[\psi\in P] \tag{S45}$$

$$\leq \mathop{\mathbb{E}}_{\psi\in\mathcal{E}}\Big[\sum_\alpha |\langle\alpha|\psi\rangle|^4\Big|\psi\notin P\Big] + \mathbb{P}_{\psi\in\mathcal{E}}[\psi\in P], \tag{S46}$$

where the notation $\mathbb{E}[\cdots\mid\cdots]$ denotes a conditional expectation value. Now we bound the first term, which is the expectation value conditioned on $\psi\notin P$, i.e., ψ not being approximately symmetric. We denote $|\psi_n\rangle = U_n|\psi\rangle$, which are approximately orthogonal states if $\psi\notin P$, $|\langle\psi_n|\psi_m\rangle| < \varepsilon$ for $n\neq m$. Moreover, observe that $|\langle\alpha|\psi_n\rangle| = |\langle\alpha|U_n|\psi\rangle| = |\langle\alpha|\psi\rangle|$, thus

$$M|\langle\alpha|\psi\rangle|^2 = \sum_{n=0}^{M-1}\langle\alpha|\psi_n\rangle\langle\psi_n|\alpha\rangle \leq \langle\alpha|\big(\tilde{\Pi}-\Pi\big)|\alpha\rangle + 1, \tag{S47}$$

where Π is the orthogonal projector into the subspace spanned by $\{|\psi_1\rangle,\ldots,|\psi_M\rangle\}$ and $\tilde{\Pi} := \sum_n |\psi_n\rangle\!\langle\psi_n|$. We now show that these two operators are close to one another for $\psi\notin P$. Specifically,

$$\langle\alpha|\big(\tilde{\Pi}-\Pi\big)|\alpha\rangle^2 \leq \|\tilde{\Pi}-\Pi\|_{\mathrm{op}}^2 \leq \|\tilde{\Pi}-\Pi\|_{\mathrm{F}}^2 = \mathrm{tr}\big((\tilde{\Pi}-\Pi)^2\big) \leq \mathrm{tr}\big(\tilde{\Pi}^2\big) - M, \tag{S48}$$

where $\|A\|_{\mathrm{F}} = \sqrt{\mathrm{tr}(AA^\dagger)}$ is the Frobenius norm, which always upper bounds the operator norm. We may compute $\mathrm{tr}\big(\tilde{\Pi}^2\big) = \sum_{n,m}|\langle\psi_n|\psi_m\rangle|^2 \leq M(M-1)\varepsilon^2 + M \leq M^2\varepsilon^2 + M$, which inserted into Eq. (S48) gives $\langle\alpha|\big(\tilde{\Pi}-\Pi\big)|\alpha\rangle \leq M\varepsilon$. Thus, from Eq. (S47) we obtain $|\langle\alpha|\psi\rangle|^2 \leq 1/M + \varepsilon$ for each α. Summing over all α, we can bound the IPR of $\psi\notin P$ as $\sum_\alpha |\langle\alpha|\psi\rangle|^4 \leq \sum_\alpha |\langle\alpha|\psi\rangle|^2(1/M+\varepsilon) = 1/M + \varepsilon$, where we used the normalization $\sum_\alpha |\langle\alpha|\psi\rangle|^2 = 1$. Averaging over $\psi\notin P$, we obtain that

$$\mathop{\mathbb{E}}_{\psi\in\mathcal{E}}\Big[\sum_\alpha |\langle\alpha|\psi\rangle|^4\Big|\psi\notin P\Big] \leq \frac{1}{M} + \varepsilon. \qquad \square$$

Lemma S8 provides a meaningful bound for the average IPR whenever P is atypical in the ensemble $\mathcal{E}$ and ε is small. We apply Lemma S8 with the unitaries U_n taken as certain translations on the lattice, which share a basis of eigenstates with the TI Hamiltonian. We select P to be the set of r-approximately periodic states with periods larger than the translations and show that $\mathbb{P}_{\psi\in\mathcal{E}}[\psi\in P]$ is exponentially small.

Let $\delta > 0$ (smaller than 1). This is the same arbitrary δ that appears in the statement of Theorem S4, which will remain fixed henceforth. We consider lattice sites $\boldsymbol{x}_1, \boldsymbol{x}_2, \ldots, \boldsymbol{x}_M \in \Lambda$ pairwise separated as $\|\boldsymbol{x}_n - \boldsymbol{x}_m\|_1 \geq L^\delta$ for all $n\neq m$. We can select at least

$$M = \left(\left\lfloor\frac{L}{\lceil L^\delta\rceil}\right\rfloor - 1\right)^D \geq \Omega(N^{1-\delta})$$

such sites (think of $\{\boldsymbol{x}_i\}_{i=1}^M$ as approximately forming a coarser sublattice). We take $U_n = \mathbb{T}^{\boldsymbol{x}_n}$, so that $\big|\langle\psi|U_m^\dagger U_n|\psi\rangle\big| \geq 2^{-r/2}$ for $n\neq m$ implies that ψ is r-approximately periodic with a period $\|\boldsymbol{n}\|_1 = \|\boldsymbol{x}_n - \boldsymbol{x}_m\|_1 \geq L^\delta$, i.e.,

$$P := \left\{\psi \,\middle|\, \exists\boldsymbol{n}\in\Lambda\colon \|\boldsymbol{n}\|_1 \geq L^\delta, |\langle\psi|\mathbb{T}^{\boldsymbol{n}}|\psi\rangle|^2 \geq 2^{-r}\right\}.$$

By Lemma S8 we obtain

$$\mathop{\mathbb{E}}_{\psi\in\mathcal{E}}\Big[\sum_j |\langle j|\psi\rangle|^4\Big] \leq O\left(\frac{1}{N^{1-\delta}}\right) + 2^{-r/2} + \mathbb{P}_{\psi\in\mathcal{E}}[\psi\in P]. \tag{S49}$$

The reason to pick increasing periods $\|\boldsymbol{n}\|_1 \geq L^\delta$ is that this will guarantee that $\mathbb{P}_{\psi\in\mathcal{E}}[\psi\in P]$ is exponentially small, as we prove next.

B. Approximately periodic states are atypical

Intuitively, periodic states with large periods will be long-range correlated, so they cannot have a significant population in an ensemble whose average state has finite correlation length. Specifically, we bound $\mathbb{P}_{\psi\in\mathcal{E}}[\psi\in P]$ by separately bounding the probability of a state having fixed period $\boldsymbol{n}$, i.e. of being in

$$P_{\boldsymbol{n}} = \left\{\psi \,\middle|\, |\langle\psi|\mathbb{T}^{\boldsymbol{n}}|\psi\rangle|^2 \geq 2^{-r}\right\}, \tag{S50}$$

and then taking the union bound over $P = \bigcup_{\|\boldsymbol{n}\|_1 \geq L^\delta} P_{\boldsymbol{n}}$.

Proposition S6 (Bound on probability of approximately periodic states)**.** *Consider an ensemble of pure states $\mathcal{E}$ with depth complexity $\mathcal{C}$, whose mean state $\rho = \mathbb{E}_{\psi\in\mathcal{E}}[|\psi\rangle\langle\psi|]$ is translation invariant and has finite correlation length ξ. Let $\boldsymbol{n} \in \Lambda$ and consider a hypercubical region A with side length ℓ and $N_A = \ell^D$ such that $\|\boldsymbol{n}\|_1 - D\ell > 2\mathcal{C}$. Then,*

$$\mathop{\mathbb{P}}_{\psi\in\mathcal{E}}[\psi \in P_{\boldsymbol{n}}] \leq 2^{r+2\mathcal{C}D\ell^{D-1}} \Big(2^{(\ell^D)} f_{\text{corr}}(2D\ell^{D-1}) e^{-(\|\boldsymbol{n}\|_1 - D\ell)/\xi} + \operatorname{tr}\big(\rho_A^2\big)\Big), \tag{S51}$$

where f_{corr} is the polynomial function from Definition S4.

Proof. Let $O_A = \bigotimes_{\boldsymbol{i}\in A} \sigma_{\boldsymbol{i}}$ be an arbitrary product of Pauli matrices $\sigma_{\boldsymbol{i}} \in \{X, Y, Z, \mathbb{1}\}$ defined over A (a so-called *Pauli string*). Let $A + \boldsymbol{n}$ denote the translation of the region A over the vector $\boldsymbol{n}$ and $O_{A+\boldsymbol{n}} = \mathbb{T}^{\boldsymbol{n}} O_A \mathbb{T}^{-\boldsymbol{n}}$. Note that the distance between A and $A+\boldsymbol{n}$ is at least $\|\boldsymbol{n}\|_1 - D\ell$. Then, because ρ has finite correlation length ξ and the number of qubits in the boundary of A is $|\partial A| = 2D\ell^{D-1}$,

$$\begin{aligned} f_{\text{corr}}(2D\ell^{D-1}) e^{-(\|\boldsymbol{n}\|_1 - D\ell)/\xi} &\geq \operatorname{tr}(\rho O_A \otimes O_{A+\boldsymbol{n}}) - \operatorname{tr}(\rho_A O_A) \operatorname{tr}(\rho_{A+\boldsymbol{n}} O_{A+\boldsymbol{n}}) &\text{(S52)}\\ &= \operatorname{tr}\Big(\Big(\mathop{\mathbb{E}}_{\psi\in\mathcal{E}}[\psi_A \otimes \psi_{A+\boldsymbol{n}}] - \rho_A \otimes \rho_{A+\boldsymbol{n}}\Big) O_A \otimes O_{A+\boldsymbol{n}}\Big), &\text{(S53)} \end{aligned}$$

where we used the short-range correlation of ψ, Lemma S4 *(ii)*, which guarantees that $\operatorname{tr}(\psi O_A \otimes O_{A+\boldsymbol{n}}) = \operatorname{tr}(\psi O_A) \operatorname{tr}(\psi O_{A+\boldsymbol{n}})$ whenever $d(A, A+\boldsymbol{n}) > 2\mathcal{C}$, which is the case here. Summing over all $4^{(\ell^D)}$ possible Pauli strings over A, and using that $\sum_{O_A} O_A \otimes O_{A+\boldsymbol{n}} = 2^{(\ell^D)} S_{A,A+\boldsymbol{n}}$, where $S_{A,A+\boldsymbol{n}}$ is the swap operator between regions A and $A + \boldsymbol{n}$, we get

$$\begin{aligned} 2^{(\ell^D)} f_{\text{corr}}(2D\ell^{D-1}) e^{-(\|\boldsymbol{n}\|_1 - D\ell)/\xi} &\geq \operatorname{tr}\Big(\Big(\mathop{\mathbb{E}}_{\psi\in\mathcal{E}}[\psi_A \otimes \psi_{A+\boldsymbol{n}}] - \rho_A \otimes \rho_{A+\boldsymbol{n}}\Big) S_{A,A+\boldsymbol{n}}\Big) &\text{(S54)}\\ &= \mathop{\mathbb{E}}_{\psi\in\mathcal{E}}[\operatorname{tr}(\psi_A \psi_{A+\boldsymbol{n}})] - \operatorname{tr}\big(\rho_A^2\big), \end{aligned}$$

where we used that $\operatorname{tr}(S(W \otimes V)) = \operatorname{tr}(WV)$ and the translation invariance $\rho_A = \rho_{A+\boldsymbol{n}}$. Now, we leverage Lemma S4 *(i)* to bound $\operatorname{tr}(\psi_A \psi_{A+\boldsymbol{n}})$ in terms of $|\langle\psi|\mathbb{T}^{\boldsymbol{n}}|\psi\rangle|^2$. Because $|\partial_{\mathcal{C}} A| = |\partial_{\mathcal{C}}(A + \boldsymbol{n})| \leq 2\mathcal{C}D\ell^{D-1}$, both ψ_A and $\psi_{A+\boldsymbol{n}}$ have rank at most $R = 2^{2\mathcal{C}D\ell^{D-1}}$, and the inequality between the trace norm and the Frobenius norm gives us

$$\operatorname{tr}(\psi_A \psi_{A+\boldsymbol{n}}) = \Big\|\sqrt{\psi_A}\sqrt{\psi_{A+\boldsymbol{n}}}\Big\|_{\text{F}}^2 \geq \frac{1}{R}\Big\|\sqrt{\psi_A}\sqrt{\psi_{A+\boldsymbol{n}}}\Big\|_{\text{tr}}^2 \geq \frac{1}{R}|\langle\psi|\mathbb{T}^{\boldsymbol{n}}|\psi\rangle|^2.$$

The last inequality follows from the fact the fidelity of reduced density matrices cannot be smaller than the global fidelity (this follows from Uhlmann's theorem) [23]. Finally, if $\psi \in P_{\boldsymbol{n}}$, then $|\langle\psi|\mathbb{T}^{\boldsymbol{n}}|\psi\rangle|^2 \geq 2^{-r}$, so

$$\mathop{\mathbb{E}}_{\psi\in\mathcal{E}}[\operatorname{tr}(\psi_A \psi_{A+\boldsymbol{n}})] \geq \frac{1}{R} \mathop{\mathbb{E}}_{\psi\in\mathcal{E}}[|\langle\psi|\mathbb{T}^{\boldsymbol{n}}|\psi\rangle|^2] \geq 2^{-(r+2\mathcal{C}D\ell^{D-1})} \mathop{\mathbb{P}}_{\psi\in\mathcal{E}}[\psi \in P_{\boldsymbol{n}}]. \tag{S55}$$

By inserting Eq. (S55) into Eq. (S54) and solving for $\mathbb{P}_{\psi\in\mathcal{E}}[\psi \in P_{\boldsymbol{n}}]$, we complete the proof. ☐

Performing a union bound over all $\|\boldsymbol{n}\|_1 \geq L^\delta$ in Eq. (S51), we obtain

$$\mathbb{P}_{\psi\in\mathcal{E}}[\psi \in P] \leq N 2^{r+2\mathcal{C}D\ell^{D-1}} \Big(2^{(\ell^D)} f_{\text{corr}}(2D\ell^{D-1}) e^{-(L^\delta - D\ell)/\xi} + \operatorname{tr}\big(\rho_A^2\big)\Big). \tag{S56}$$

The term $\operatorname{tr}\big(\rho_A^2\big)$ is the subsystem purity of ρ, which by condition S4 is exponentially small in $N_A = \ell^D$, i.e., $\operatorname{tr}\big(\rho_A^2\big) \leq e^{-\Omega(\ell^D)}$. Thus, from Eq. (S56) we get the asymptotic bound on the probability of a state being r-approximately periodic:

$$\mathbb{P}_{\psi\in\mathcal{E}}[\psi \in P] \leq N 2^{r+O(\mathcal{C}\ell^{D-1})} \Big(\operatorname{poly}(\ell) e^{O(\ell^D)} e^{-\Omega(L^\delta)} + e^{-\Omega(\ell^D)}\Big), \tag{S57}$$

where $\operatorname{poly}(\ell)$ denotes a polynomial on ℓ.

Inserting Eq. (S57) into Eq. (S49) gives the bound on the IPR,

$$\mathop{\mathbb{E}}_{\psi\in\mathcal{E}}\Big[\sum_j |\langle j|\psi\rangle|^4\Big] \leq O\Big(\frac{1}{N^{1-\delta}}\Big) + \underbrace{N \operatorname{poly}(\ell) e^{O(\ell^D) - \Omega(L^\delta) + O(\mathcal{C}\ell^{D-1}) + O(r)}}_{(\star)} + \underbrace{N e^{O(r) + O(\mathcal{C}\ell^{D-1}) - \Omega(\ell^D)}}_{(\star\star)} + \underbrace{e^{-\Omega(r)}}_{(\star\star\star)}. \tag{S58}$$

We are free to select how ℓ and r scale with $L = N^{1/D}$. We select the scaling so the first term on the right-hand side of Eq. (S58) dominates over the other three terms. Specifically, we pick

$$\ell = \Theta(L^a) \qquad \text{and} \qquad r = \Theta(L^b) \tag{S59}$$

so $(\star\star\star) = e^{-\Omega(L^b)}$. Furthermore, because the complexity grows subpolynomially, $\mathcal{C} \leq O(L^\nu)$ for any $\nu > 0$, we have $\mathcal{C}\ell^{D-1} \leq O(L^{(D-1)a+\nu})$. Thus, as long as $0 < b < aD < \delta$ and $\nu < a$, we have $(D-1)a + \nu < aD < \delta$ and consequently $(\star) \leq e^{-\Omega(L^\delta)} \leq e^{-\Omega(L^b)}$ and $(\star\star) \leq e^{-\Omega(L^{aD})} \leq e^{-\Omega(L^b)}$.

All in all, the last three terms on the right-hand side of Eq. (S58) are upper bounded by $e^{-\Omega(L^b)}$, over which the first term dominates. Then we may conclude,

$$\mathop{\mathbb{E}}_{\psi\in\mathcal{E}}\left[\sum_j |\langle j|\psi\rangle|^4\right] \leq O\left(\frac{1}{N^{1-\delta}}\right), \tag{S60}$$

which is the statement of Theorem S4.

V. THERMALIZATION TO GENERALIZED GIBBS STATES

In this section we extend our proof of quantum thermalization beyond the canonical ensemble $g_\beta \propto e^{-\beta H}$. We consider systems with additional conserved quantities where the general form of the equilibrium state has additional structure, for example a grand canonical ensemble or a generalized Gibbs ensemble [24]. Specifically, we consider a set of local conserved quantities Q_i, meaning that $[H, Q_i] = 0$ and Q_i can can be decomposed as a sum of observables with bounded operator norm and each acting on a geometrically local region. The generalized Gibbs state (GGS) is parametrized by a vector $\boldsymbol{\lambda} = (\lambda_1, \lambda_2, \ldots, \lambda_m)$ of *generalized chemical potentials* (GCP) as

$$g_{\boldsymbol{\lambda}} = \frac{\exp\Big(-\sum_{j=1}^m \lambda_j Q_j\Big)}{\mathrm{tr}\Big(\exp\Big(-\sum_{j=1}^m \lambda_j Q_j\Big)\Big)}. \tag{S61}$$

For example if we have energy conservation $Q_1 = H$ and one single additional $U(1)$ symmetry (e.g., $Q_2 = \sum_{\boldsymbol{j}\in\Lambda} Z_{\boldsymbol{j}}$), the state $g_{(\lambda_1=\beta,\lambda_2=-\beta\mu)}$ describes a grand canonical ensemble with chemical potential μ. If the system is integrable and thus supports an extensive number of conserved quantities, there may be a chemical potential associated to each of them.

To state our main result on thermalization to the GGS, we first need to generalize our notion of MEE.

Definition S10 (Generalized maximally entropic ensemble (G-MEE))**.** Given a vector of GCPs $\boldsymbol{\lambda}$, we say an ensemble of pure states $\mathcal{E}_{\boldsymbol{\lambda}}$ has GCPs $\boldsymbol{\lambda}$ if the average expectation value of the conserved quantities are equal to those of the GGS,

$$\forall j : \mathop{\mathbb{E}}_{\psi\in\mathcal{E}_{\boldsymbol{\lambda}}}[\langle\psi|Q_j|\psi\rangle] = \mathrm{tr}(g_{\boldsymbol{\lambda}} Q_j). \tag{S62}$$

Furthermore, an ensemble $\mathcal{E}^*_{\boldsymbol{\lambda},\mathcal{C}}$ with GCPs $\boldsymbol{\lambda}$ and complexity $\mathcal{C}$ is a G-MEE if it maximizes the von Neumann entropy of its average state among all ensembles with the same GCPs and complexity,

$$\mathcal{E}^*_{\boldsymbol{\lambda},\mathcal{C}} \in \mathop{\mathrm{argmax}}_{\mathcal{E}_{\boldsymbol{\lambda},\mathcal{C}}} S(\rho_{\boldsymbol{\lambda},\mathcal{C}}). \tag{S63}$$

We establish a proof of quantum thermalization to GGSs. The GCPs play the role of inverse temperature β in Theorem 1, so the statement about thermalization at high temperature (or equivalently small β) is here promoted to a statement about small GCPs, in the sense that each entry of $\boldsymbol{\lambda}$ is below a threshold constant.

Theorem S5 (Quantum thermalization to generalized Gibbs states)**.** *Consider any translation invariant, geometrically local Hamiltonian H for N qubits in D-dimensional lattice with nondegenerate spectral gaps. Let $Q_1, \ldots, Q_m$ be a constant number of local translation invariant conserved quantities $[Q_i, H] = 0$. There exists a threshold constant $\lambda_* > 0$ such that for any $\boldsymbol{\lambda} = (\lambda_1, \ldots, \lambda_m)$ with generalized chemical potentials bounded as $|\lambda_i| \leq \lambda_*$, any G-MEE with shallow complexity $\mathcal{E}_{\boldsymbol{\lambda},\mathcal{C}}$ thermalizes,*

$$\mathop{\mathbb{E}}_{\psi\in\mathcal{E}_{\boldsymbol{\lambda},\mathcal{C}}} \mathop{\mathbb{E}}_t[\|\psi(t) - g_{\boldsymbol{\lambda}}\|_A] \leq O\big(N^{-1/2+\delta}\big) \tag{S64}$$

for any geometrically local region A and for any $\delta > 0$.

Before moving on to explain the proof, two remarks are in order. First, note that Theorem S5 considers a constant number of conserved quantities m which should be independent of N. Nevertheless, Theorem S5 still applies to the dynamics generated by a Hamiltonian with more local conserved quantities, such as Bethe ansatz integrable systems. We note, however, that in such systems one can only impose a constant number of nonzero GCPs for the ensemble of initial states. As we will see below, the threshold GCP λ_* depends on m and on the choice of $Q_1, \ldots, Q_m$.

The second remark is that, like Theorem 1, Theorem S5 requires the nondegenerate gap condition, which also requires no degeneracies in the spectrum. In particular, we require the Hamiltonian to be free of any nonabelian symmetries (which necessarily imply degeneracy). This has two consequences: first, all the conserved quantities must commute pairwise $[Q_i, Q_j] = 0$, and secondly, the system cannot possess a spatial inversion symmetry (which does not commute with the translation operator). The latter consequence somewhat hinders the applicability of Theorem S5 to certain interacting integrable spin chains, such as the Heisenberg or XXZ/XYZ models which usually posses a spatial inversion symmetry. Nevertheless we note that one can construct integrable spin chains without inversion symmetry[3], to which Theorem S5 applies. It is an interesting and important direction to extend our results to systems with nonabelian symmetries.

The proof of Theorem S5 follows the same path as Theorem 1. We prove a generalized EDGE (G-EDGE) Theorem, and then we show that any shallow-complexity G-MEE at low GCP is a G-EDGE.

A. Properties of the generalized Gibbs state

We begin by proving some properties of the GGS.

Proposition S7 (Properties of generalized Gibbs states at low chemical potential)**.** *Let $Q_1, Q_2, \ldots Q_m$ be local observables, where m is a constant. Let $\boldsymbol{\lambda} = (\lambda_1, \ldots, \lambda_m)$ be any GCP vector.*

(a) [Maximum entropy principle]. Among all states ρ satisfying the constraints $\mathrm{tr}(\rho Q_i) = W^{(i)}_{\boldsymbol{\lambda}}$, where $W^{(i)}_{\boldsymbol{\lambda}} := \mathrm{tr}(g_{\boldsymbol{\lambda}} Q_i)$, the GGS $g_{\boldsymbol{\lambda}}$ uniquely maximizes the von Neumann entropy

$$g_{\boldsymbol{\lambda}} = \underset{\rho : \mathrm{tr}(\rho Q_i) = W^{(i)}_{\boldsymbol{\lambda}}}{\mathrm{argmax}} \; S(\rho). \tag{S65}$$

(b) [Finite correlation length]. There exists a positive constant $\lambda_*^{(\mathrm{corr})}$ such that $g_{\boldsymbol{\lambda}}$ has finite correlation length whenever $\max_i |\lambda_i| \le \lambda_*^{(\mathrm{corr})}$.

(c) [Separability]. There exists a positive constant $\lambda_*^{(\mathrm{sep})}$ such that $g_{\boldsymbol{\lambda}}$ is separable whenever $\max_i |\lambda_i| \le \lambda_*^{(\mathrm{sep})}$.

(d) [Small subsystem purity]. If $\max_i |\lambda_i| \le \lambda_*^{(\mathrm{corr})}$, then $g_{\boldsymbol{\lambda}}$ has an exponentially small subsystem purity, $\mathrm{tr}\big((g_{\boldsymbol{\lambda},A})^2\big) \le e^{-\Omega(N_A)}$ for any region A with N_A qubits.

Proof. Property (a) follows from a standard argument: First, we compute the relative entropy

$$D(\rho || g_{\boldsymbol{\lambda}}) = -S(\rho) + \sum_i \lambda_i \, \mathrm{tr}(Q_i \rho) + \log\Big(\mathrm{tr}\Big(e^{-\sum_i \lambda_i Q_i}\Big)\Big). \tag{S66}$$

Second, we impose the constraints $\mathrm{tr}(\rho Q_i) = W^{(i)}_{\boldsymbol{\lambda}}$, and thus we see than maximizing $S(\rho)$ is equivalent to minimizing the relative entropy, whose unique minimum $D(\rho||g_{\boldsymbol{\lambda}}) = 0$ is attained by $\rho = g_{\boldsymbol{\lambda}}$.

For properties (b), (c), and (d) the idea is simply to write $g_{\boldsymbol{\lambda}}$ as a regular Gibbs state for some local Hamiltonian $\widetilde{H}$ and inverse temperature $\widetilde{\beta}$, and leverage the corresponding properties that we already established for the Gibbs state at high temperature. Specifically, we define

$$\widetilde{H}_{\boldsymbol{\lambda}} = \frac{1}{\max_i |\lambda_i|} \sum_{i=1}^{m} \lambda_i Q_i \tag{S67}$$

and $\widetilde{\beta}_{\boldsymbol{\lambda}} = \max_i |\lambda_i|$ so that immediately $\widetilde{g}_{\widetilde{\beta}_{\boldsymbol{\lambda}}} := \exp(-\widetilde{\beta}_{\boldsymbol{\lambda}} \widetilde{H}_{\boldsymbol{\lambda}}) / \mathrm{tr}(\exp(-\widetilde{\beta}_{\boldsymbol{\lambda}} \widetilde{H}_{\boldsymbol{\lambda}})) = g_{\boldsymbol{\lambda}}$. Furthermore, the Hamiltonian $\widetilde{H}_{\boldsymbol{\lambda}}$ is local, because m is a constant and each Q_i is local.

---

[3] We thank Nicholas O'Dea for pointing this out to us.

We begin by proving separability. If we apply Lemma S1 to $\widetilde{H}_{\boldsymbol{\lambda}}$ we obtain a constant inverse temperature $\widetilde{\beta}_*^{(\mathrm{sep})} > 0$ such that for any $\widetilde{\beta} \leq \widetilde{\beta}_*^{(\mathrm{sep})}$, the state $\widetilde{g}_{\widetilde{\beta}}$ is separable. The key point to note is that the constant $\widetilde{\beta}_*^{(\mathrm{sep})}$ is actually independent of $\boldsymbol{\lambda}$ (for generic choices such that $\lambda_i \neq 0$, which we assume without loss of generality). This is because the separability threshold $\widetilde{\beta}_*^{(\mathrm{sep})}$ only depends on the locality properties of the Hamiltonian $\widetilde{H}_{\boldsymbol{\lambda}}$, and is independent of the specific relative weights of its terms. Thus, we can define $\lambda_*^{(\mathrm{sep})} := \widetilde{\beta}_*^{(\mathrm{sep})}$. Then, for any $\boldsymbol{\lambda}$ such that $\widetilde{\beta}_{\boldsymbol{\lambda}} := \max_i |\lambda_i| \leq \lambda_*^{(\mathrm{sep})}$, we have $\widetilde{\beta}_{\boldsymbol{\lambda}} \leq \widetilde{\beta}_*^{(\mathrm{sep})}$, so $g_{\boldsymbol{\lambda}} = \widetilde{g}_{\widetilde{\beta}_{\boldsymbol{\lambda}}}$ is separable.

We note that, in general, $\widetilde{\beta}_*^{(\mathrm{sep})}$ depends on the locality properties of $\widetilde{H}_{\boldsymbol{\lambda}}$ such as the number of terms acting on a certain qubit. For our purposes, this translates to that the associated $\lambda_*^{(\mathrm{sep})}$ depends on the number of conserved quantities m.

The argument for finite correlation length is the same, by now applying Lemma S1 to $\widetilde{H}_{\boldsymbol{\lambda}}$. The exponentially small subsystem purity follows from Proposition S2, applied to $g_{\boldsymbol{\lambda}} = \widetilde{g}_{\widetilde{\beta}}$.

□

B. Generalized EDGE theorem

Now, we generalize the EDGE theorem. For this we need to generalize the concept of EDGEs.

Definition S11 (Generalized energy dispersed Gibbs ensemble)**.** An ensemble of states $\mathcal{E}_{\boldsymbol{\lambda}}$ is a generalized EDGE (G-EDGE) if it satisfies the following two conditions:

(a) [Generalized Gibbs ensemble]. The average of the ensemble is close to $g_{\boldsymbol{\lambda}}$,

$$\left\| \mathop{\mathbb{E}}_{\psi \in \mathcal{E}_{\boldsymbol{\lambda}}} [\psi] - g_{\boldsymbol{\lambda}} \right\| \leq 2^{-N} \varepsilon_{\mathrm{GE}}(N). \tag{S68}$$

(b) [Energy dispersion]. The average IPR in the energy eigenbasis is upper bounded as

$$\mathop{\mathbb{E}}_{\psi \in \mathcal{E}_{\boldsymbol{\lambda}}} \left[\sum_{j=1}^{2^N} |\langle E_j | \psi \rangle|^4 \right] \leq \varepsilon_{\mathrm{ED}}(N). \tag{S69}$$

We can prove a G-EDGE theorem:

Theorem S6 (Generalized EDGE)**.** *Let H be a TI, geometrically local Hamiltonian with nondegenerate spectral gaps on a D-dimensional periodic lattice with N qubits and $\mathcal{E}_{\boldsymbol{\lambda}}$ be an G-EDGE. Assume that the generalized Gibbs state $g_{\boldsymbol{\lambda}}$ has finite correlation length. Then $\mathbb{E}_{\psi \in \mathcal{E}_{\boldsymbol{\lambda}}} \mathbb{E}_{t \geq 0} [\|g_{\boldsymbol{\lambda}} - \psi(t)\|_A]$ is upper bounded by the right-hand side of Eq* (S21)*. At sufficiently small chemical potential $\max_i |\lambda_i| < \lambda_*^{(\mathrm{con})}$, we can take any $\gamma < 1/2$.*[4]

Proof. The proof is the same as the EDGE Theorem S2, with the only difference that the equivalence of temporal and thermal ensembles now reads

$$\mathop{\mathbb{E}}_{\psi \in \mathcal{E}_{\boldsymbol{\lambda}}} [\|\rho_\infty - g_{\boldsymbol{\lambda}}\|_A] = O(N^{-\gamma}) + 2\varepsilon_{\mathrm{GE}}(N). \tag{S70}$$

We prove this in the same manner as Proposition S4, simply replacing β by $\boldsymbol{\lambda}$. Note that Corollary S1 also applies to $g_{\boldsymbol{\lambda}}$ because this state is also diagonal in the eigenbasis of H, as $[Q_i, H] = 0$. Furthermore, to obtain the improved scaling $\gamma < 1/2$, we simply write $g_{\boldsymbol{\lambda}}$ as a high-temperature Gibbs state (as in the proof of S7), and the constant $\lambda_*^{(\mathrm{con})}$ is obtained in the same manner as the constants in Proposition S7, from the threshold temperature $\beta_*^{(\mathrm{con})}$ of Proposition S3. □

---

[4] Note that a finite-time statement analogous to Theorem S3 can also be proven.

C. Shallow-complexity G-MEEs are G-EDGEs at low generalized chemical potential

Finally, we prove that any G-MEE of shallow-complexity is an G-EDGE. We first have an analogous result to Lemma S7, which establishes property (a) of a G-EDGE with $\varepsilon_{\rm GE} = 0$.

Lemma S9 (G-MEE average to generalized Gibbs states at low generalized chemical potential.)**.** *Let $Q_1, Q_2, \ldots Q_m$ be local observables, where m is a constant. For any complexity function $\mathcal{C}(N)$, there exists a positive constant $\lambda_*^{(\mathcal{C})} \geq \lambda_*^{(\rm sep)}$ such that for any chemical potential vector $\boldsymbol{\lambda} = (\lambda_1, \ldots, \lambda_m)$ with $\max_i |\lambda_i| \leq \lambda_*^{(\mathcal{C})}$, any G-MEE $\mathcal{E}_{\boldsymbol{\lambda},\mathcal{C}}$ averages to the generalized Gibbs state,*

$$\mathop{\mathbb{E}}_{\psi \in \mathcal{E}_{\boldsymbol{\lambda},\mathcal{C}}} [\psi] = g_{\boldsymbol{\lambda}}. \tag{S71}$$

The proof is the same as Lemma S7, replacing β with $\boldsymbol{\lambda}$ and leveraging the maximum entropy principle and separability at low GCPs proven in Proposition S7 (a) and (c), respectively.

Property (b) of a G-EDGE follows simply from Theorem 3. The GGS at low $\boldsymbol{\lambda}$, i.e. with $\max_j |\lambda_j| \leq \lambda_* := \min\{\lambda_*^{(\rm sep)}, \lambda_*^{(\rm corr)}, \lambda_*^{(\rm con)}\}$ is TI whenever each Q_i is TI, and has finite correlation length and an exponentially small subsystem purity by Proposition S7 (b) and (d), respectively. Thus, by Theorem 3, we obtain a bound on the average IPR $\varepsilon_{\rm ED} = O(N^{-1+\delta})$, which combined with Lemma S9 and the G-EDGE Theorem S6 gives us Theorem S5.

REFERENCES

[1] D. K. Mark, F. Surace, A. Elben, A. L. Shaw, J. Choi, G. Refael, M. Endres, and S. Choi, Maximum entropy principle in deep thermalization and in Hilbert-space ergodicity, Phys. Rev. X **14**, 041051 (2024).

[2] Y. Huang, Extensive entropy from unitary evolution (2021), 2104.02053 [quant-ph].

[3] Y. Huang and A. W. Harrow, Instability of localization in translation-invariant systems (2019), 1907.13392 [cond-mat.dis-nn].

[4] M. P. Müller, E. Adlam, L. Masanes, and N. Wiebe, Thermalization and canonical typicality in translation-invariant quantum lattice systems, Comm. Math. Phys. **340**, 499–561 (2015).

[5] H. Araki, Gibbs states of a one dimensional quantum lattice, Communications in Mathematical Physics **14**, 120–157 (1969).

[6] A. Bluhm, A. Capel, and A. Pérez-Hernández, Exponential decay of mutual information for Gibbs states of local Hamiltonians, Quantum **6**, 650 (2022).

[7] M. Kliesch, C. Gogolin, M. J. Kastoryano, A. Riera, and J. Eisert, Locality of temperature, Phys. Rev. X **4**, 031019 (2014).

[8] A. Bakshi, A. Liu, A. Moitra, and E. Tang, High-temperature Gibbs states are unentangled and efficiently preparable, in *2024 IEEE 65th Annual Symposium on Foundations of Computer Science (FOCS)* (2024) pp. 1027–1036.

[9] Y. Huang, Random product states at high temperature equilibrate exponentially well (2024), arXiv:2409.08436 [cond-mat.stat-mech].

[10] G. Biroli, C. Kollath, and A. M. Läuchli, Effect of rare fluctuations on the thermalization of isolated quantum systems, Phys. Rev. Lett. **105**, 250401 (2010).

[11] T. Mori, Weak eigenstate thermalization with large deviation bound (2016), arXiv:1609.09776 [cond-mat.stat-mech].

[12] F. G. S. L. Brandão, E. Crosson, M. B. Şahinoğlu, and J. Bowen, Quantum error correcting codes in eigenstates of translation-invariant spin chains, Phys. Rev. Lett. **123**, 110502 (2019).

[13] A. M. Alhambra, J. Riddell, and L. P. García-Pintos, Time evolution of correlation functions in quantum many-body systems, Phys. Rev. Lett. **124**, 110605 (2020).

[14] N. Linden, S. Popescu, A. J. Short, and A. Winter, Quantum mechanical evolution towards thermal equilibrium, Phys. Rev. E **79**, 061103 (2009).

[15] M. Srednicki, Thermal fluctuations in quantized chaotic systems, Journal of Physics A: Mathematical and General **29**, L75–L79 (1996).

[16] Y. Huang, High-precision simulation of finite-size thermalizing systems at long times (2024), arXiv:2406.05399 [cond-mat.stat-mech].

[17] T. Kuwahara and K. Saito, Gaussian concentration bound and Ensemble equivalence in generic quantum many-body systems including long-range interactions, Annals of Physics **421**, 168278 (2020).

[18] A. Anshu, Concentration bounds for quantum states with finite correlation length on quantum spin lattice systems, New Journal of Physics **18**, 083011 (2016).

[19] H. Wilming, T. R. de Oliveira, A. J. Short, and J. Eisert, Equilibration times in closed quantum many-body systems, in *Thermodynamics in the Quantum Regime: Fundamental Aspects and New Directions*, edited by F. Binder, L. A. Correa, C. Gogolin, J. Anders, and G. Adesso (Springer International Publishing, Cham, 2018) pp. 435–455.

[20] A. J. Short and T. C. Farrelly, Quantum equilibration in finite time, New Journal of Physics **14**, 013063 (2012).

[21] A. S. L. Malabarba, L. P. García-Pintos, N. Linden, T. C. Farrelly, and A. J. Short, Quantum systems equilibrate rapidly for most observables, Phys. Rev. E **90**, 012121 (2014).

[22] S. Balasubramanian, S. Gopalakrishnan, A. Khudorozhkov, and E. Lake, Glassy word problems: Ultraslow relaxation, Hilbert space jamming, and computational complexity, Phys. Rev. X **14**, 021034 (2024).

[23] M. A. Nielsen and I. L. Chuang, *Quantum Computation and Quantum Information* (Cambridge University Press, Cambridge, England, 2010).
[24] L. Vidmar and M. Rigol, Generalized Gibbs ensemble in integrable lattice models, Journal of Statistical Mechanics: Theory and Experiment **2016**, 064007 (2016).
