# Supplementary material for: Quantum thermalization must occur in translation-invariant systems at high temperature
Source: Nat Commun. 2025 Dec 16;17:75. doi: 10.1038/s41467-025-66777-7 (PMC12770369; doi:10.1038/s41467-025-66777-7)
Supplement: Supplementary file 2 — Transparent Peer Review file [file 41467_2025_66777_MOESM2_ESM.pdf]

nature portfolio

Peer Review File

# Quantum thermalization must occur in translation-invariant systems at high temperature

Corresponding Author: Professor Soonwon Choi

This manuscript has been previously reviewed at another journal that is not operating a transparent peer review scheme. The manuscript was considered suitable for publication without further review at Nature Communications.

**Open Access** This Peer Review File is licensed under a Creative Commons Attribution 4.0 International License, which permits use, sharing, adaptation, distribution and reproduction in any medium or format, as long as you give appropriate credit to the original author(s) and the source, provide a link to the Creative Commons license, and indicate if changes were made.
In cases where reviewers are anonymous, credit should be given to 'Anonymous Referee' and the source.
The images or other third party material in this Peer Review File are included in the article's Creative Commons license, unless indicated otherwise in a credit line to the material. If material is not included in the article's Creative Commons license and your intended use is not permitted by statutory regulation or exceeds the permitted use, you will need to obtain permission directly from the copyright holder.
To view a copy of this license, visit https://creativecommons.org/licenses/by/4.0/
